# Genome-Wide Identification, Characterization and Phylogenetic Analysis of 50 Catfish ATP-Binding Cassette (ABC) Transporter Genes

**DOI:** 10.1371/journal.pone.0063895

**Published:** 2013-05-16

**Authors:** Shikai Liu, Qi Li, Zhanjiang Liu

**Affiliations:** 1 The Fish Molecular Genetics and Biotechnology Laboratory, Aquatic Genomics Unit, Department of Fisheries and Allied Aquacultures and Program of Cell and Molecular Biosciences, Auburn University, Auburn, Alabama, United States of America; 2 The Shellfish Genetics and Breeding Laboratory, Fisheries College, Ocean University of China, Qingdao, P.R. China; University of Lausanne, Switzerland

## Abstract

**Background:**

Although a large set of full-length transcripts was recently assembled in catfish, annotation of large gene families, especially those with duplications, is still a great challenge. Most often, complexities in annotation cause mis-identification and thereby much confusion in the scientific literature. As such, detailed phylogenetic analysis and/or orthology analysis are required for annotation of genes involved in gene families. The ATP-binding cassette (ABC) transporter gene superfamily is a large gene family that encodes membrane proteins that transport a diverse set of substrates across membranes, playing important roles in protecting organisms from diverse environment.

**Methodology/Principal Findings:**

In this work, we identified a set of 50 ABC transporters in catfish genome. Phylogenetic analysis allowed their identification and annotation into seven subfamilies, including 9 ABCA genes, 12 ABCB genes, 12 ABCC genes, 5 ABCD genes, 2 ABCE genes, 4 ABCF genes and 6 ABCG genes. Most ABC transporters are conserved among vertebrates, though cases of recent gene duplications and gene losses do exist. Gene duplications in catfish were found for ABCA1, ABCB3, ABCB6, ABCC5, ABCD3, ABCE1, ABCF2 and ABCG2.

**Conclusion/Significance:**

The whole set of catfish ABC transporters provide the essential genomic resources for future biochemical, toxicological and physiological studies of ABC drug efflux transporters. The establishment of orthologies should allow functional inferences with the information from model species, though the function of lineage-specific genes can be distinct because of specific living environment with different selection pressure.

## Introduction

Fish are exposed to numerous toxicants present in the aquatic environment. Catfish, as a bottom-dwelling fish species, is highly adaptable to its associated aquatic environment such as low dissolved oxygen, high level of toxins (e.g., hydrogen sulfide, ammonia and nitrite) and various xenobiotics. Therefore, catfish can serve as a good research model for toxicological studies. Numerous genomic resources have been developed to study detoxification-related genes in catfish, including a large number of ESTs [1], [2], draft whole genome sequences (unpublished), and RNA-Seq transcriptome assemblies [3]–[6]. We recently assembled a comprehensive transcriptome and generated over 14,000 full-length transcripts by RNA-Seq of a doubled-haploid channel catfish [4]. Such full-length transcripts allowed genome-wide identification and annotation of gene families in catfish. In this work, we conducted a study to identify and characterize a superfamily of ATP-binding cassette (ABC) transporters, the main efflux pumps that could be involved in detoxification pathways.

The ATP-binding cassette (ABC) transporters are one of the largest protein families and are present in all organisms from bacteria to human [7]–[10]. Prototypical ABC transporters are membrane-bound proteins coupling ATP hydrolysis to transport substrates across biological membranes including ions, sugars, amino acids, polypeptides, toxic metabolites, and xenobiotics [11], [12]. All ABC transporters share a highly conserved domain architecture. A functional transporter requires the combination of two ATP-binding domains (also known as nucleotide-binding domains, NBDs) and two transmembrane domains (TMDs).

Eukaryotic ABC transporters are either full transporters combining all required domains in one polypeptide (2 NBDs and 2 TMDs), or half transporters consisting of one NBD and one TMD that need to form homo- or heterodimer to generate a functional pump [11], [12]. ABC transporters are classified into seven (A–G) or eight (A–H) subfamilies based on their primary sequence and domain structures and organization [8]–[10], [13]. Based on their functions, the ABC transporters can be classified as exporters, importers, and non-transport proteins [14]. The exporters and importers play roles in transporting a wide variety of substances, while the third class of ABC transporters (ABCE and ABCF) is not involved in molecule transport because they possess only two NBDs but lack TMDs.

The ABC transporter family from animals was first characterized in the human with a total of 48 members [10]. In humans, mutations of many ABC genes have been associated with hereditary diseases, including cystic fibrosis (CF), adrenoleukodystrophy (ALD) and cholesterol metabolism disorders [11], [12], [15], [16]. In worms and insects, several members of the ABC transporters have been reported to play roles in drug/insecticide resistance [16], . Due to the importance of ABC transporters, extensive investigations have been conducted in numerous species [10], [13], [18]–[23], but studies in fish species have been limited to model species such as zebrafish [8]. Here we report the identification, characterization, and phylogenetic analysis of a set of 50 ABC transporters in catfish.

## Results and Discussion

### Identification and Phylogenetic Analysis of ABC Transporters in Catfish

**Figure 1:**
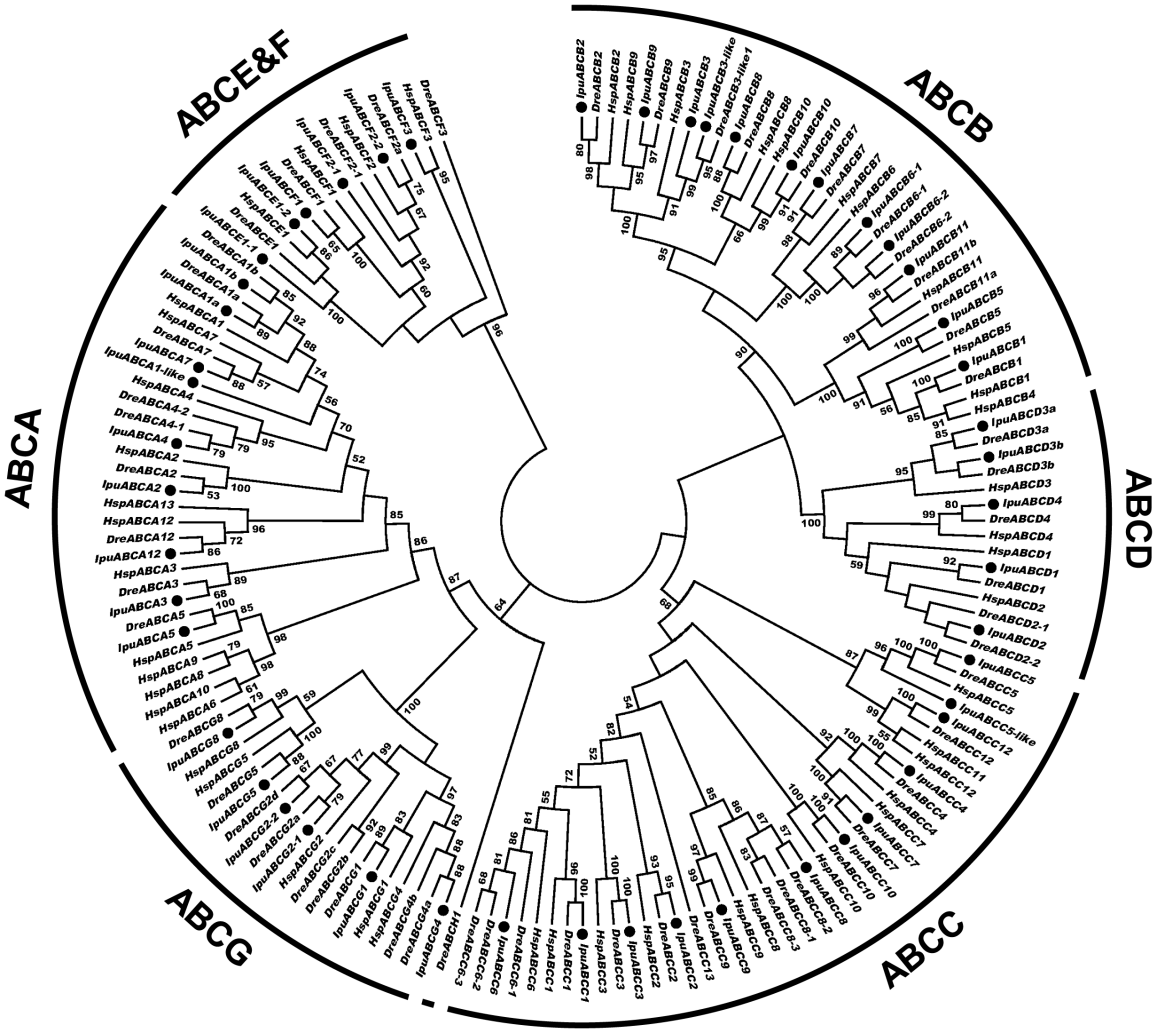
Subfamilies of the catfish ABC transporters. The phylogenetic tree was constructed using maximum likelihood algorithm under the JTT+I+G model of amino acid substitution as described in detail in Material and method section. Numbers around the nodes correspond to bootstrap support values in percentages. Accession numbers for all sequences are provided in Table S1. Abbreviations: Ipu, *Ictalurus punctatus*; Dre, *Danio rerio* and Hsp, *Homo sapiens*. The black dots indicate catfish ABC genes.

A total of 50 ABC transporter genes were identified in catfish genome. Their transcripts, coding sequences, domain structures and accession numbers are summarized in Table 1 and Figure S1. The 50 transporters were divided into seven subfamilies according to phylogenetic analysis including 9 ABCAs, 12 ABCBs, 12 ABCCs, 5 ABCDs, 2 ABCEs, 4 ABCFs and 6 ABCGs (Figure 1, Figure S1; Figure S2). Detailed phylogenetic analyses and in some cases syntenic analyses were conducted for each subfamily as described below.

**Table 1:** Summary of 50 ABC transporter genes identified in catfish genome.

Gene symbol	mRNA (bp)	CDS (aa)	Domain structure	CDS status	Accession
**ABCA1a**	8612	2268	(6/7TMD-NBD)2	Complete	JT414963
**ABCA1b**	13391	2283	(6TMD-NBD)2	Complete	JT444833
**ABCA1-like**	7737	2230	(6/7TMD-NBD)2	Complete	JT339739
**ABCA2**	9734	2503	(8/5TMD-NBD)2	Complete	JT473783
**ABCA3**	8325	1710	(7TMD-NBD)2	Complete	JT416806
**ABCA4**	5222	1826	4TMD-?-6TMD-NBD	Partial	JT348097
**ABCA5**	6337	1653	(7/6TMD-NBD)2	Complete	JT417197
**ABCA7**	5864	1722	5TMD-NBD-3TMD-?	Partial	JT445101
**ABCA12**	5846	1828	(5TMD-NBD)2-?	Partial	JT400726
**ABCB1**	4217	1318	(6/5TMD-NBD)2	Complete	JT312655
**ABCB2**	4193	735	7TMD-NBD	Complete	JT415437
**ABCB3**	6171	718	6TMD-NBD	Complete	JT478417
**ABCB3-like**	2287	710	4TMD-NBD	Complete	JT449254
**ABCB5**	4574	1344	(5/6TMD-NBD)2	Complete	JT410848
**ABCB6-1**	3769	869	9TMD-NBD	Complete	JT415190
**ABCB6-2**	2828	660	6TMD-NBD	Complete	JT175332
**ABCB7**	5178	742	5TMD-NBD	Complete	JT415109
**ABCB8**	2595	707	5TMD-NBD	Complete	JT410950
**ABCB9**	3460	787	7TMD-NBD	Complete	JT415528
**ABCB10**	4265	665	5TMD-NBD	Complete	JT412222
**ABCB11**	4624	1348	(5/4TMD-NBD)2	Complete	JT412475
**ABCC1**	4436	1477	5TMD-(6/5TMD-NBD)2	Complete	JT422444&JT445381
**ABCC2**	5920	1565	5TMD-(4/3TMD-NBD)2	Complete	JT406352
**ABCC3**	5849	1537	5TMD-(4/5TMD-NBD)2	Complete	JT418649
**ABCC4**	8287	1329	(6/5TMD-NBD)2	Complete	JT415334
**ABCC5**	4394	1423	(6/5TMD-NBD)2	Complete	JT416749
**ABCC5-like**	6226	1403	(5/4TMD-NBD)2	Complete	JT427226
**ABCC6**	2423	768	5TMD-(5/?TMD-?)2	Partial	JT444514
**ABCC7**	5757	1479	(4/6TMD-NBD)2	Complete	JT410330
**ABCC8**	7622	1580	5TMD-(6/4TMD-NBD)2	Complete	JT409716
**ABCC9**	7356	1569	5TMD-(6/3TMD-NBD)2	Complete	JT414362
**ABCC10**	5667	1544	5TMD-(6/5TMD-NBD)2	Complete	JT412570
**ABCC12**	4848	1388	(6/5TMD-NBD)2	Complete	JT406166
**ABCD1**	7248	779	?TMD-NBD	Complete	JT409589
**ABCD2**	1064	354	?TMD-NBD	Partial	JT280165
**ABCD3a**	3084	659	4TMD-NBD	Complete	JT405596
**ABCD3b**	2242	660	3TMD-NBD	Complete	JT452399
**ABCD4**	4689	604	3TMD-NBD	Complete	JT405742
**ABCE1-1**	3018	599	NBD-NBD	Complete	JT412510
**ABCE1-2**	2187	599	NBD-NBD	Complete	JT199283
**ABCF1**	3092	863	NBD-NBD	Complete	JT467213
**ABCF2-1**	2734	610	NBD-NBD	Complete	JT417384
**ABCF2-2**	2166	609	NBD-NBD	Complete	JT406901
**ABCF3**	2811	710	NBD-NBD	Complete	JT409535
**ABCG1**	1518	534	NBD-3TMD-?	Partial	JT391455
**ABCG2-1**	4231	637	NBD-5TMD	Complete	JT244049
**ABCG2-2**	2734	650	NBD-7TMD	Complete	JT410422
**ABCG4**	3797	644	NBD-7TMD	Complete	JT413745
**ABCG5**	4110	655	NBD-6TMD	Complete	JT407113
**ABCG8**	6105	679	NBD-6TMD	Complete	JT413428

#### ABCA subfamily

Nine ABCA genes were identified in the catfish genome including ABCA1a, ABCA1b, ABCA1-like, ABCA2, ABCA3, ABCA4, ABCA5, ABCA7, and ABCA12. Full-length coding sequences were obtained for six of the nine catfish ABCA transporters except ABCA4, ABCA7 and ABCA12. All catfish ABCA proteins are full transporters, even though some of the sequences are partial (Table 1 and Figure S1).

**Figure 2:**
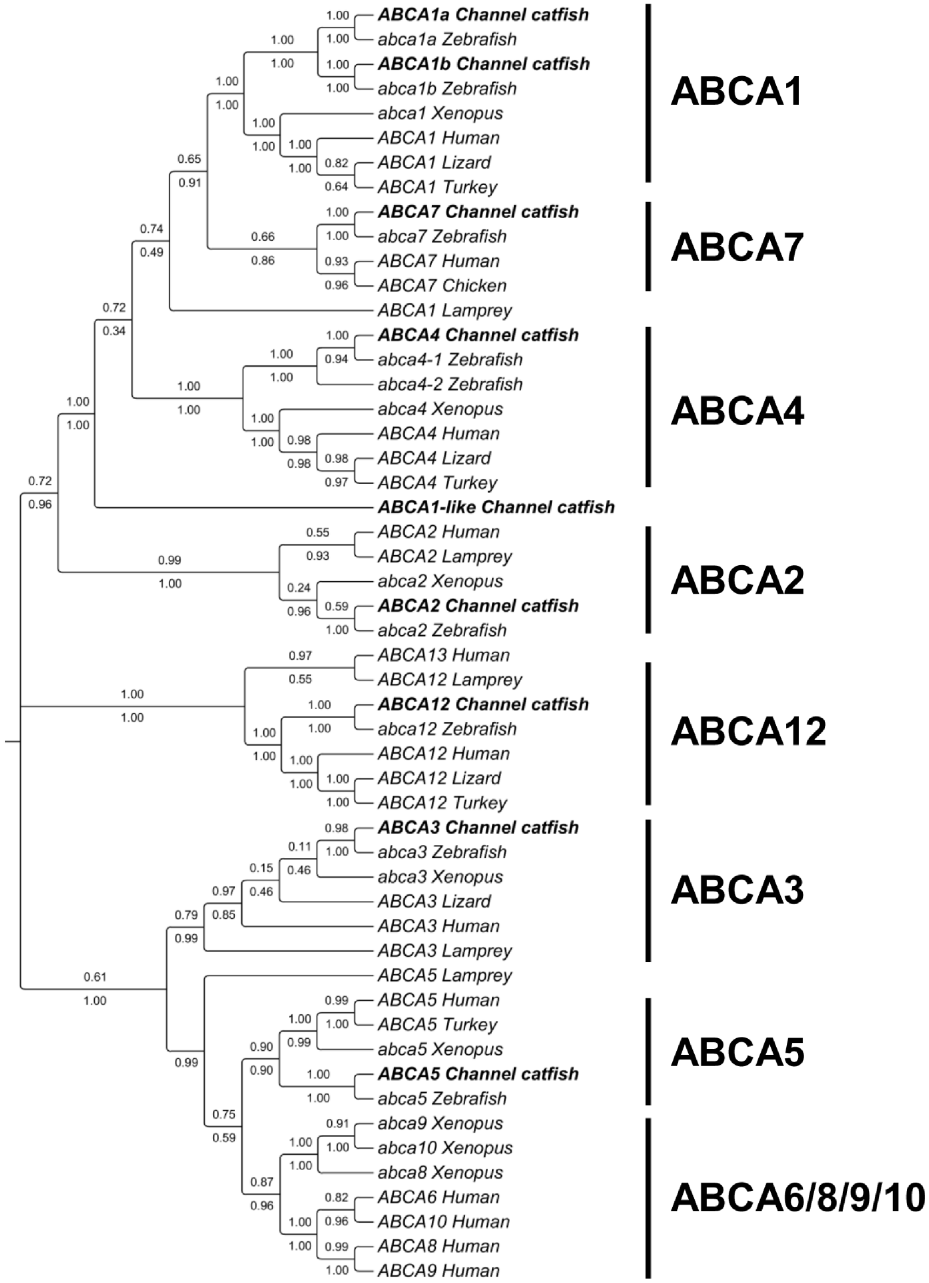
Phylogenetic tree of ABCA subfamily transporters. The phylogenetic tree was obtained as in Figure 1. Numbers around the nodes correspond to bootstrap support values (1.0, i.e., 100%). Accession numbers for all sequences are provided in Table S1.

The phylogenetic analysis supported the annotation of catfish ABCA genes. Each of catfish ABCA genes clustered with its respective counterpart from other species (Figure 2 and Figure S2). The catfish ABCA1a and ABCA1b group together with zebrafish abca1a and abca1b, respectively, and form one clade with ABCA1 genes from other species. The ABCA1-like gene was placed into a separate clade (Figure 2), though the blast search supported it as ABCA1 related genes with a high level of similarities. The ABCA1 and ABCA7 are closely related as indicated by the phylogenetic tree. Based on the ABCA1 from lamprey, a very ancient lineage of vertebrate, ABCA7 appears to be derived from ABCA1 by duplication events. The catfish ABCA4 groups together with one of the two ABCA4 in zebrafish, forming ABCA4 clade with other ABCA4 genes. In the monophyletic clade containing ABCA5, the catfish ABCA5 groups together with other ABCA5 genes, but no corresponding complements exist for ABCA6, ABCA8, ABCA9 and ABCA10 (Figure 2). In human, ABCA6, ABCA8, ABCA9 and ABCA10 are clustered with ABCA5 on chromosome 17q24 [8]. Similarly, three clustered genes are found in chicken [8] as well as in *Xenopus* (Figure 2) [24]. This indicates that expansion of the ABCA5-related genes occurred after the split of the teleost fish [25]. As indicated by the phylogenetic analysis, the ABCA5 is conserved throughout all vertebrates, while the non-ABCA5 genes in the cluster are expanded in a lineage-specific pattern (Figure 2).

#### ABCB subfamily

**Figure 3:**
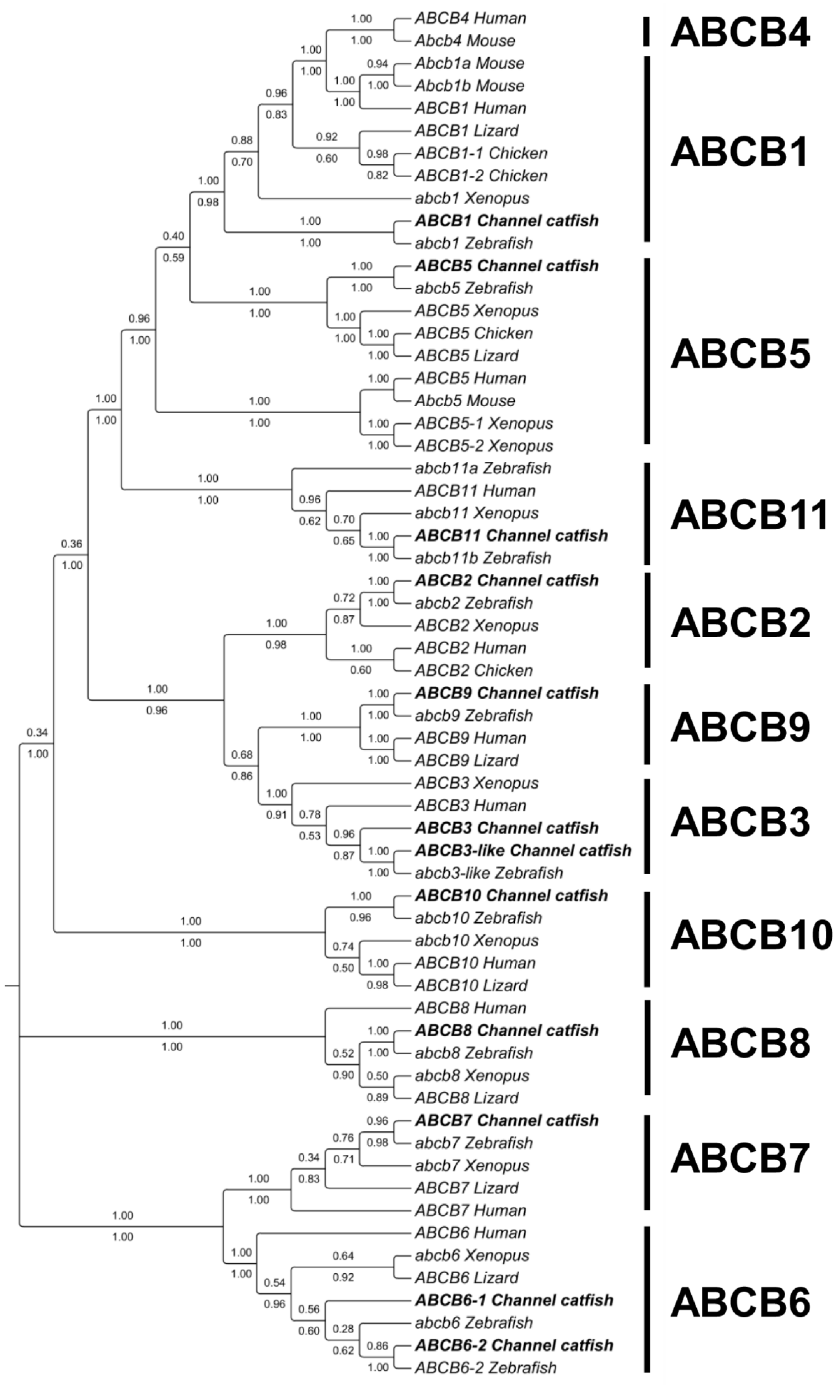
Phylogenetic tree of ABCB subfamily transporters. The phylogenetic tree was obtained as in Figure 1. Numbers around the nodes correspond to bootstrap support values (1.0, i.e., 100%). Accession numbers for all sequences are provided in Table S1.

A total of 12 ABCB transporters were identified in catfish including ABCB1, ABCB2, ABCB3, ABCB3-like, ABCB5, ABCB6-1, ABCB6-2, ABCB7, ABCB8, ABCB9, ABCB10, and ABCB11. Compared to the 11 ABCB transporters in humans, only ABCB4 was not found in catfish (see below), but two duplicates were identified with ABCB3 and ABCB6 (Figure 3). Of the 12 ABCB transporters, three are full transporters and nine are half transporters. (Table 1 and Figure S1).

**Figure 4:**
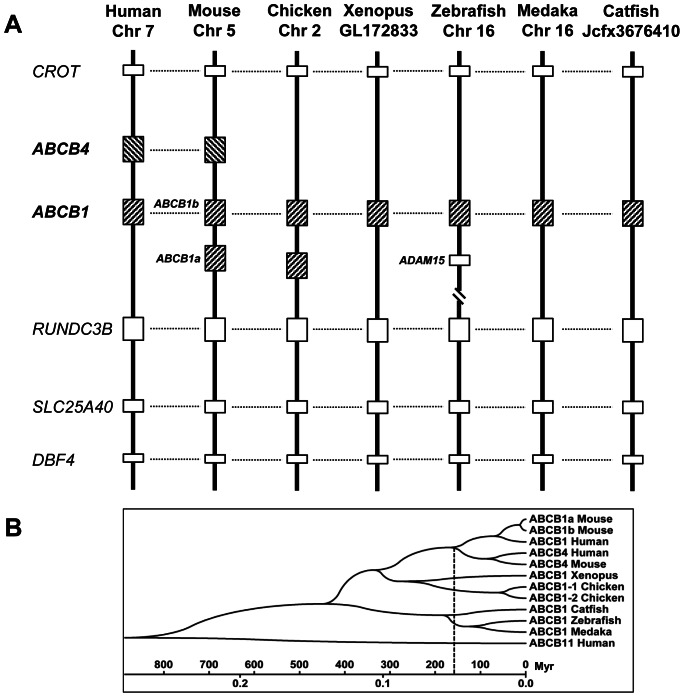
Analysis of conserved synteny blocks harboring ABCB1/4 gene in several vertebrates (A) and dating of gene duplication events (B). (A) Horizontal lines denote orthologous relationships. Abbreviations: CROT, carnitine O-octanoyltransferase; ABCB4, ATP-binding cassette, sub-family B (MDR/TAP), member 4; ABCB1, ATP-binding cassette, sub-family B (MDR/TAP), member 1; RUNDC3B, RUN domain containing 3B; SLC25A40, solute carrier family 25, member 40 and DBF4, DBF4 homolog (*S. cerevisiae*). (B) Date estimates for the duplication to create ABCB4 genes in mammals. The duplication time was estimated at about 170 million years (Myr) ago as indicated by dashed line.

All the catfish ABCB transporters were placed into distinct clades well supported by phylogenetic analysis with the exception of ABCB1 and ABCB5 (Figure 3). As shown in Figure 3, the catfish ABCB1 fell into a subclade with the zebrafish ABCB1, but they did not fall into the clade as expected with all the other ABCB1s from other species. Therefore, phylogenetic analysis alone did not provide a solid support for the annotation of the catfish ABCB1. We then conducted syntenic analysis to provide insight into the orthologies of these related genes. As shown in Figure 4, it is apparent that the annotation of the catfish ABCB1 was supported by the conserved syntenies. In the genomic neighborhood containing the ABCB1 gene, the gene order was well conserved, with DBF4, SLC25A40, and RUNDC3B on one side of the ABCB1 gene, and CROT gene on the other side of the ABCB1 gene in the genomes of human, mouse, chicken, Xenopus, zebrafish, medaka, and catfish. Apparently, the ABCB1 and ABCB4 in the human and mouse genomes are co-orthologous to ABCB1 in the fish genomes (Figure 4).

**Figure 5:**
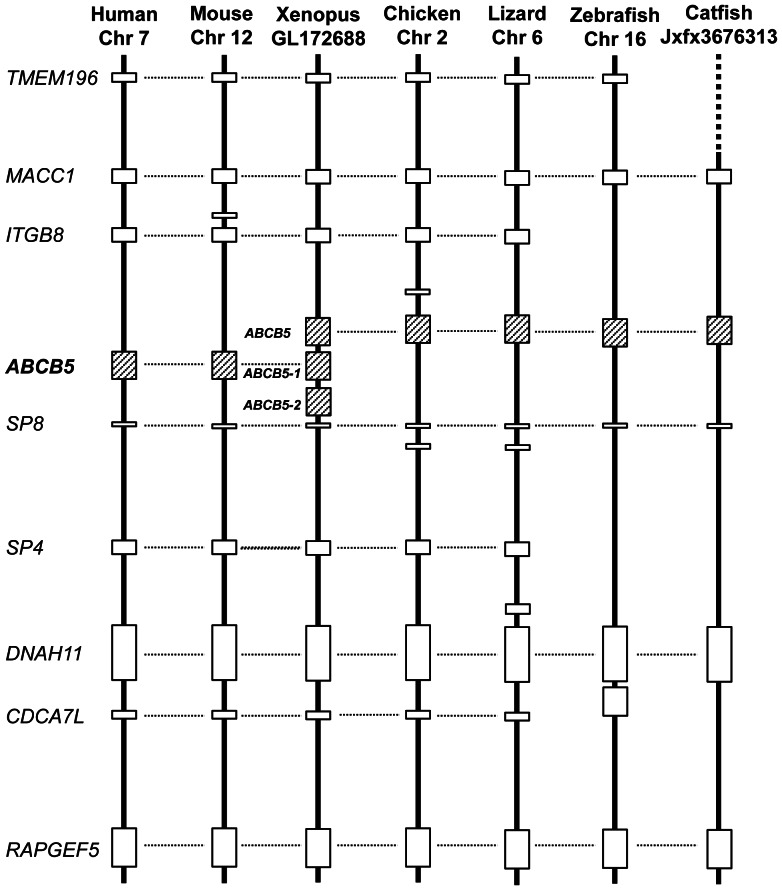
Analysis of synteny blocks harboring ABCB5 gene in several vertebrates. Horizontal lines denote orthologous relationships. Abbreviations: TMEM196, transmembrane protein 196; MACC1, metastasis associated in colon cancer 1; ITGB8, integrin, beta 8; ABCB5, ATP-binding cassette, sub-family B (MDR/TAP), member 5; SP8, Sp8 transcription factor; SP4, Sp4 transcription factor; DNAH11, dynein, axonemal, heavy chain 11; CDCA7L, cell division cycle associated 7-like and RAPGEF5, Rap guanine nucleotide exchange factor (GEF) 5.

Similarly, phylogenetic analysis did not provide a concrete support for the annotation of ABCB5. On the one hand, the catfish ABCB5 fell into the same clade containing ABCB5 of zebrafish, Xenopus, chicken, and lizard, but on the other hand, these ABCB5s did not fall into the same clade containing the mammalian ABCB5s as well as two additional ABCB5s from Xenopus (Figure 3). We therefore conducted syntenic analysis to determine the orthologies of these related genes. As shown in Figure 5, gene contents in the genome neighborhood varied a little, but the conserved syntenies are still obvious, suggesting the orthologous relationship among all the ABCB5 genes.

The ABCB1, ABCB4 and ABCB5 are closely related, which share a common ancestor in the history of chordates [26]. The ABCB1 gene is reported to have undergone a duplication to create ABCB4 and a separate duplication to generate Abcb1b gene in rodents and opossum [27]. Specifically, Moitra et al. (2011) proposed that a lineage-specific gene duplication in human that resulted in the birth of ABCB1 and ABCB4, which occurred after the split of mammals from reptiles [26]. Therefore, ABCB1 is the ancestral gene and ABCB4 exists exclusively in mammalian genomes [8], [27].

To determine the age of duplication for ABCB4, we adopted a molecular clock test with the protein sequences of ABCB1 and ABCB4 using the human ABCB11 as an outgroup. The branch-length test indicated that the sequences have evolved at similar rates (p<5%, Z test), therefore, a linearized tree was constructed (Figure 4B). The tree was calibrated using a mammal-fish split of 450 million years (Myr) and rodent-primate split date of 75 Myr [28]. Both estimates agree with each other and give the duplication date for ABCB4 in mammals as ∼170 Myr, which is after the split of mammals from birds and reptiles ∼350 Myr (Figure 4B). Taken together, we believe it is reasonable to annotate the catfish gene as ABCB1 because it’s orthologous to mammalian ABCB1 and ABCB4 but is not ABCB4. It is noteworthy that ABCB1 genes in some non-mammals may have been mistakenly annotated as “ABCB4”.

#### ABCC subfamil**y**

**Figure 6:**
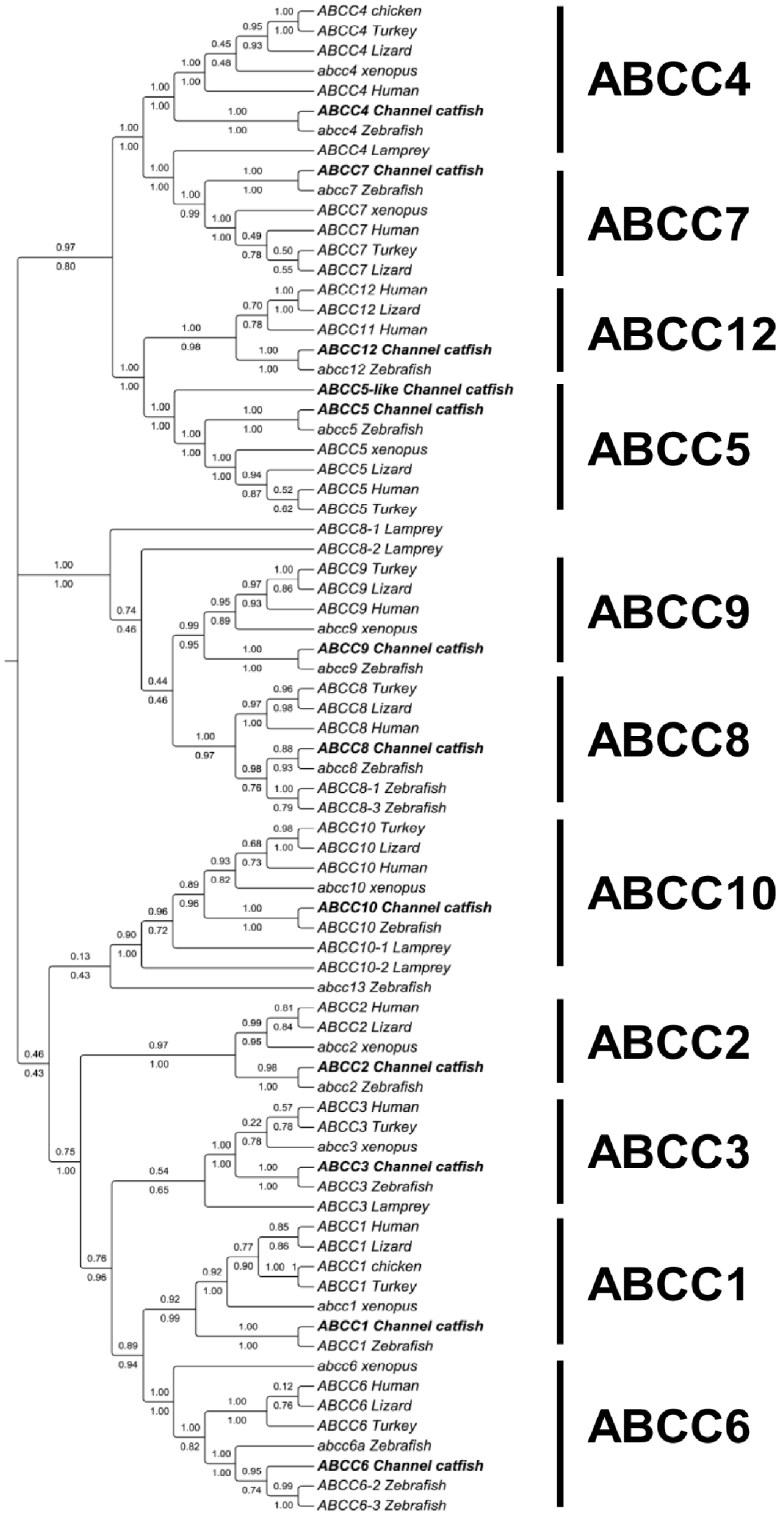
Phylogenetic tree of ABCC subfamily transporters. The phylogenetic tree was obtained as in Figure 1. Numbers around the nodes correspond to bootstrap support values (1.0, i.e., 100%). Accession numbers for all sequences are provided in Table S1.

Twelve ABCC genes were identified in the catfish genome including ABCC1, ABCC2, ABCC3, ABCC4, ABCC5, ABCC5-like, ABCC6, ABCC7, ABCC8, ABCC9, ABCC10, and ABCC12 (Table 1 and Figure S1). In general, phylogenetic analysis well supported the annotations of catfish ABCC genes (Figure 6 and S2). All the catfish ABCC genes fell into their respective clades with orthologous genes from other species. The majority of multi-drug resistance proteins (MRPs) formed a monophyletic clade with five sub-clades (ABCC1, 2, 3, 6 and 10) while ABCC4/ABCC5 fell under a different clade containing ABCC7 and ABCC12 (Figure 6). According to the phylogenetic tree, ABCC7 is related to ABCC4, while ABCC12 is related to ABCC5.

The ABCC subfamily consists of transporters known as multidrug resistance-associated proteins (MRPs) which transport diverse substrates including drugs, endogenous compounds and xenobiotics. In addition to MRPs, the ABCC subfamily includes the chloride channel CFTR and the sulfonylurea receptors (SUR1 and SUR2) [10], [29]. The human ABCC subfamily consists of 12 full transporters [8]: MRPs 1–6 (ABCC1–6) and MRPs 7–9 (ABCC10–12), CFTR (ABCC7), SUR1 (ABCC8) and SUR2 (ABCC9) [8], [10], [30]. Of these, no orthologue of ABCC11 was identified from catfish. In addition, ABCC13 was not found in catfish, but was reported from zebrafish, dog, and macaque [30], [31].

#### ABCD subfamily

The ABCD transporters are located to the peroxisome and are involved in the transport of fatty acids and/or fatty acyl-CoAs into peroxisome [32]–[34]. There are four ABCD members in human: ABCD1–4. We identified all the four homologs in catfish genome. The four catfish ABCDs are all half transporters with only one NBD and one TMD domain, similar to ABCDs from other species (Table 1 and Figure S1).

**Figure 7:**
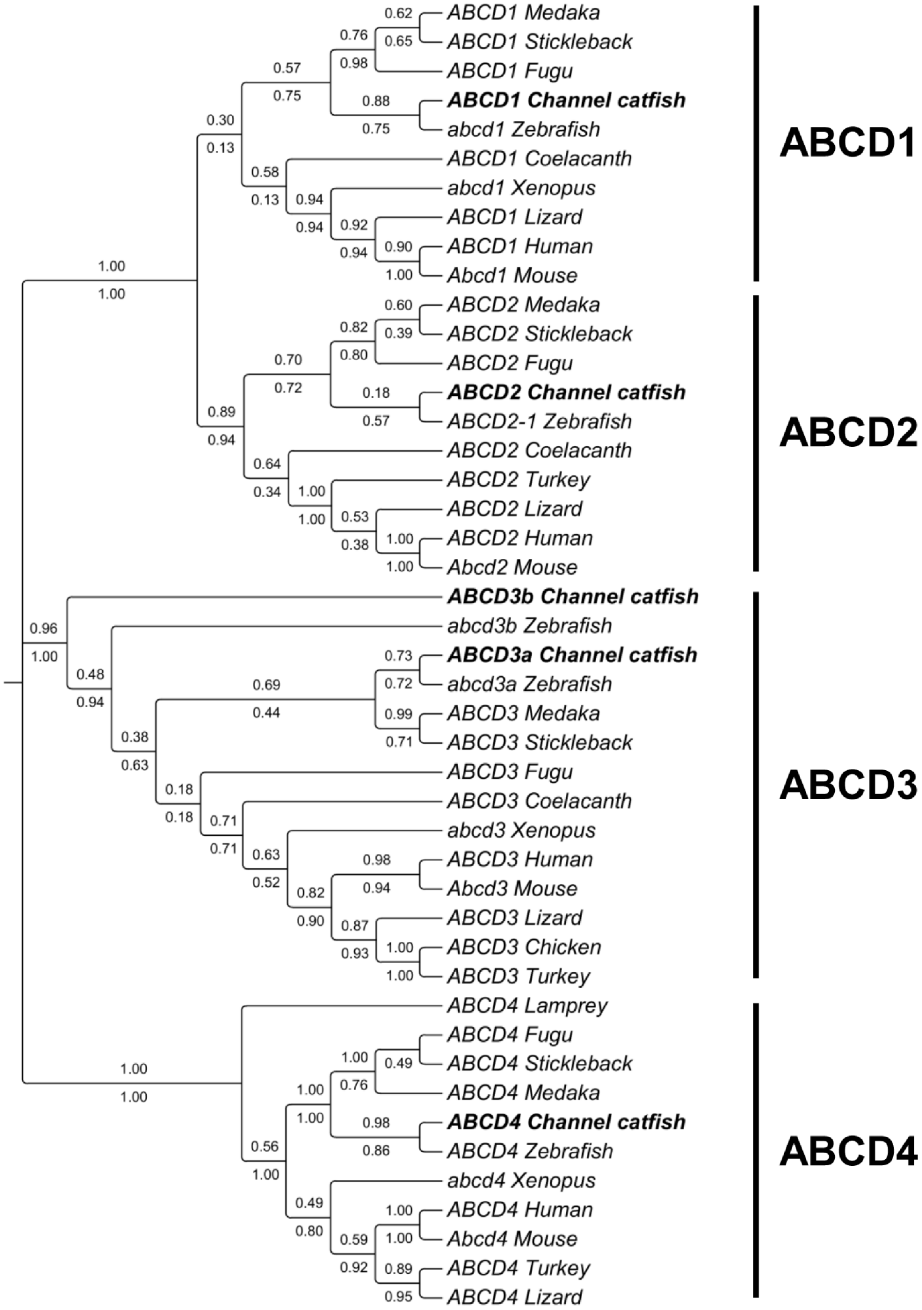
Phylogenetic tree of ABCD subfamily transporters. The phylogenetic tree was obtained as in Figure 1. Numbers around the nodes correspond to bootstrap support values (1.0, i.e., 100%). Accession numbers for all sequences are provided in Table S1.

All genes in the ABCD subfamily are highly conserved. As revealed by phylogenetic analysis, four major clades of ABCDs were formed with one catfish member in each clade except ABCD3 that included two catfish ABCD3s (Figure 7 and S2). The phylogenetic tree well support the annotation of ABCD1, ABCD2, ABCD3a, ABCD4. However, there was an additional ABCD3-like gene in catfish and zebrafish. Following the nomenclature of zebrafish with this duplicated ABCD3-like gene, we also named the catfish ABCD3-like gene ABCD3b, but the orthology needs to be determined.

#### ABCE and ABCF subfamily

**Figure 8:**
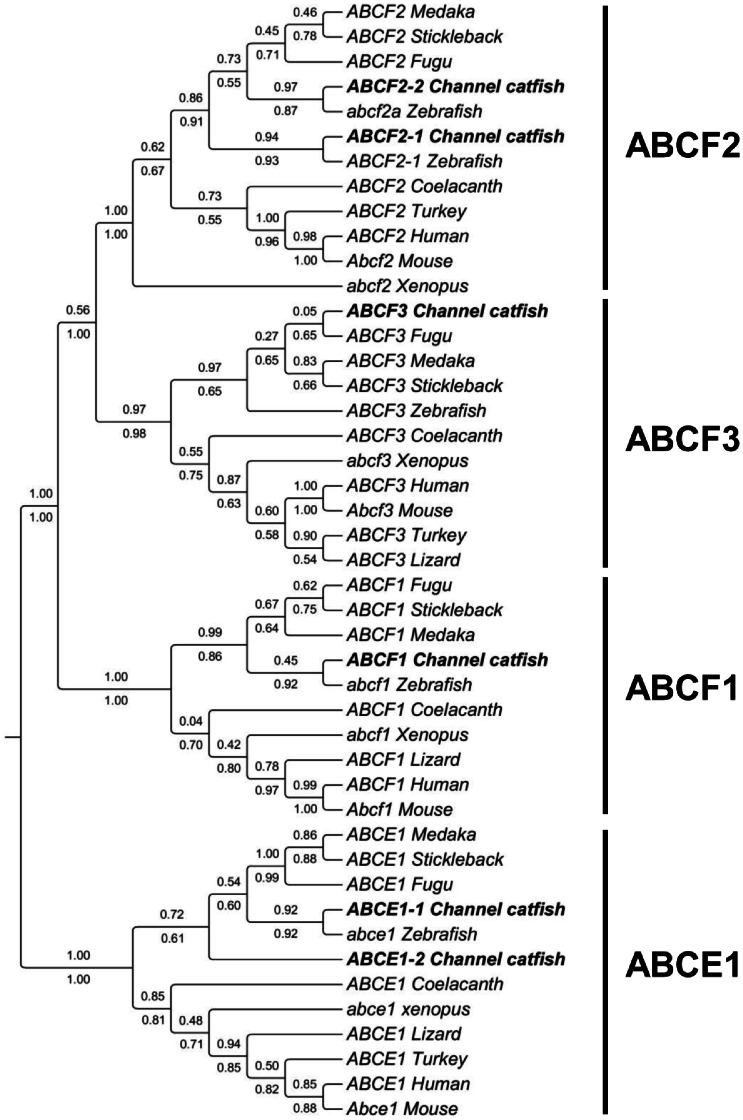
Phylogenetic tree of ABCE and ABCF subfamily transporters. The phylogenetic tree was obtained as in Figure 1. Numbers around the nodes correspond to bootstrap support values (1.0, i.e., 100%). Accession numbers for all sequences are provided in Table S1.

The ABCE and ABCF subfamilies consist of genes that possess two NBDs but no TMDs, making them non-functional as transporters. The ABCE subfamily contains a single gene in human, but two ABCE genes were identified in catfish genome. There are three ABCF genes in human: ABCF1, ABCF2 and ABCF3. All ABCF homologs were identified in catfish genome. Phylogenetic analysis well supported the annotation of these genes. However, the catfish ABCF2 is duplicated in the catfish genome as is in the zebrafish genome, whereas the catfish ABCE1 is uniquely duplicated (Figure 8).

#### ABCG subfamily

**Figure 9:**
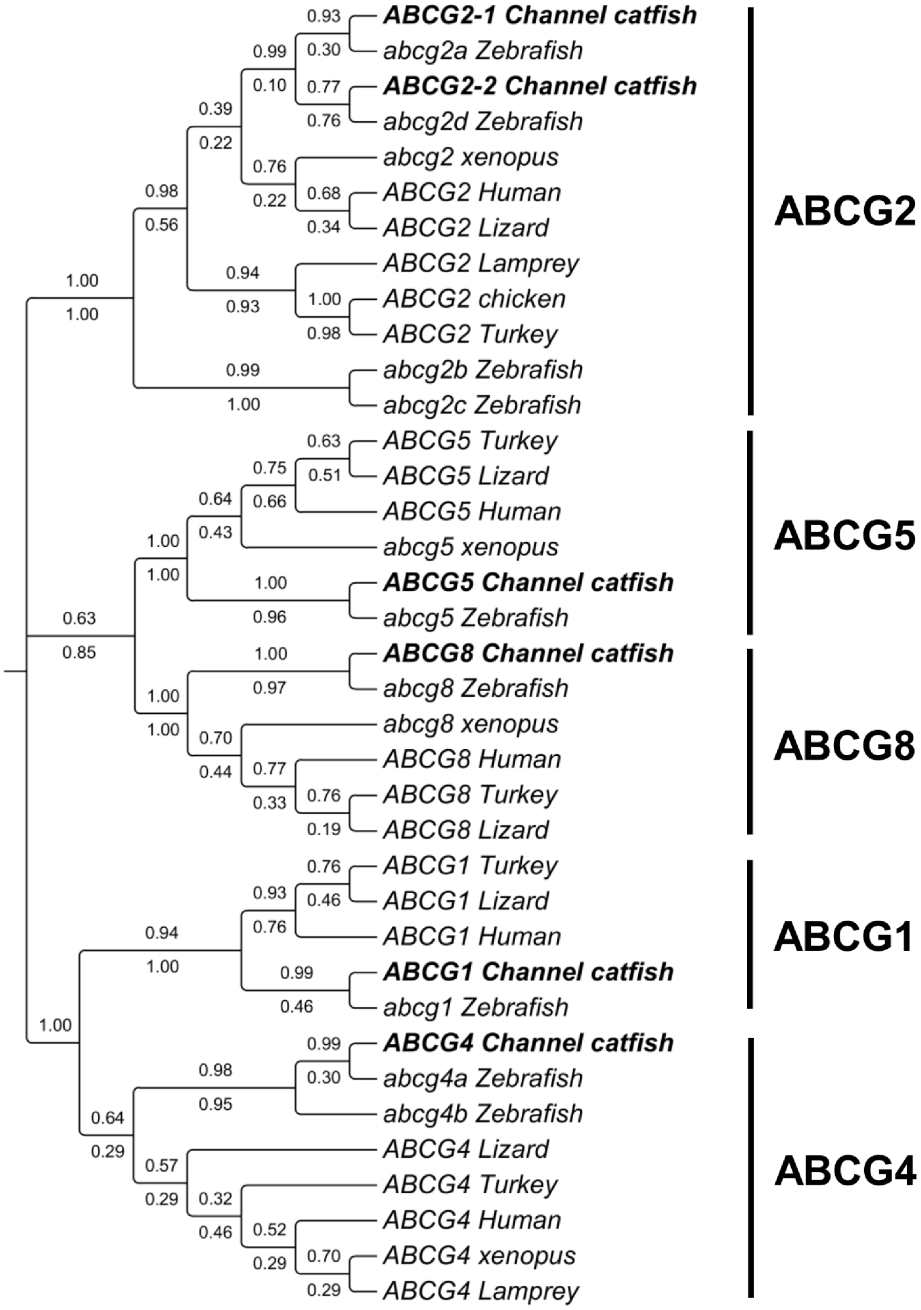
Phylogenetic tree of ABCG subfamily transporters. The phylogenetic tree was obtained as in Figure 1. Numbers around the nodes correspond to bootstrap support values (1.0, i.e., 100%). Accession numbers for all sequences are provided in Table S1.

The human ABCG subfamily contains five members: ABCG1, ABCG2, ABCG4, ABCG5 and ABCG8. All ABCG transporters in metazoans are half transporters. In contrast to other half transporters, ABCGs show distinct domain structure with TMDs being located at the C-terminus of the NBDs. We identified all the ABCG homologs in the catfish genome. The phylogenetic analysis as shown in Figure 9 well supported the annotations of the catfish ABCGs. The catfish ABCG2 is duplicated as it is in zebrafish, with strong phylogenetic evidence of their orthologous relations.

### Gene Duplications and Losses of ABC Transporters in Catfish

Teleost fish constitute over half of all vertebrate species and have adapted to a variety of marine and freshwater habitats [35]. The high diversification of teleost fish is proposed to correlate with the fish-specific genome duplication (3R), which is estimated to occur around 226–350 Myr ago [36], [37]. As a result of genome duplication, teleost fish have two paralogous copies for many genes, while only one ortholog is present in tetrapods [37]. Followed by whole genome duplication, lineage-specific paralog duplication and loss are frequently observed during evolution [38], [39]. Gene duplication and loss produces an enormous number of new genes with the potential for partitioned functions or neofunctions.

Several members of ABC transporters in catfish have undergone gene duplications. These genes include ABCA1, ABCB3, ABCB6, ABCC5, ABCD3, ABCE1, ABCF2 and ABCG2 (Table 1). In addition to the duplication, lineage-specific gene losses were observed as well. The copy numbers of ABC genes in several vertebrate genomes were investigated (Table 2), with focus on catfish and other fish with sequenced genomes. It’s apparent that ABCA1, ABCA4, ABCB6, ABCC4, ABCC6, ABCG2 and ABCG4 were duplicated as a result of fish-specific genome duplication because two or more copies of respective gene were simultaneously present in the examined teleost fish. Of which, single copy of ABCA4, ABCC4, ABCC6 and ABCG4 were identified in catfish genome suggesting potential gene losses after whole genome duplication. The ABCA6, ABCA8, ABCA9, ABCA10, ABCA12, ABCA13, ABCA14, ABCA15, ABCA17, ABCB4, ABCC11 and ABCG3 were not found in any fish genome. Such non-fish ABC genes appeared as a result of duplications after the split of tetrapods from teleost fish [8], [27]. Of which, ABCA6, ABCA8, ABCA9, ABCA10, ABCA12, ABCA13 and ABCB4 were found only in mammals, and ABCA14, ABCA15 and ABCA17 were found only in rodents and dog, ABCC11 was found in only human while ABCG3 was found only in rodents [8].

**Table 2:** Comparison of ABC transporters in several vertebrate genomes*.

	Human	Mouse	Catfish	Zebrafish	Medaka	Fugu	Stickleback	Tetraodon	Tilapia	Cod	Coelacanth
***ABCA1***	*1*	*1*	*(3)*	*2*	*2*	*2*	*2*	*2*	*2*	*2*	*1*
**ABCA2**	1	1	1	1	0	0	0	0	0	0	0
**ABCA3**	1	1	1	1	1	1	1	1	1	1	1
***ABCA4***	*1*	*1*	*1*	*2*	*2*	*2*	*2*	*1*	*2*	*2*	*1*
**ABCA5**	1	1	1	1	0	1	1	1	1	1	1
**ABCA6**	**1**	**1**	**0**	**0**	**0**	**0**	**0**	**0**	**0**	**0**	**0**
**ABCA7**	1	1	1	1	1	1	1	1	0	1	1
**ABCA8**	**1**	**2**	**0**	**0**	**0**	**0**	**0**	**0**	**0**	**0**	**0**
**ABCA9**	**1**	**1**	**0**	**0**	**0**	**0**	**0**	**0**	**0**	**0**	**0**
**ABCA10**	**1**	**0**	**0**	**0**	**0**	**0**	**0**	**0**	**0**	**0**	**0**
**ABCA12**	1	1	1	1	1	1	1	0	1	1	1
**ABCA13**	**1**	**1**	**0**	**0**	**0**	**0**	**0**	**0**	**0**	**0**	**0**
**ABCA14**	**0**	**1**	**0**	**0**	**0**	**0**	**0**	**0**	**0**	**0**	**0**
**ABCA15**	**0**	**1**	**0**	**0**	**0**	**0**	**0**	**0**	**0**	**0**	**0**
**ABCA17**	**0**	**1**	**0**	**0**	**0**	**0**	**0**	**0**	**0**	**0**	**0**
**ABCB1**	1	2	1	1	1	0	1	0	1	1	0
**ABCB2**	1	1	1	1	1	1	1	1	1	1	1
**ABCB3**	1	1	(2)	1	0	0	0	0	0	0	0
**ABCB4**	**1**	**1**	**0**	**0**	**0**	**0**	**0**	**0**	**0**	**0**	**0**
**ABCB5**	1	1	1	1	0	0	0	0	0	0	1
***ABCB6***	*1*	*1*	*(2)*	*2*	*1*	*2*	*2*	*2*	*2*	*1*	*1*
**ABCB7**	1	1	1	1	1	1	1	1	1	1	1
**ABCB8**	1	1	1	1	1	1	1	1	1	1	1
**ABCB9**	1	1	1	1	1	1	1	1	0	1	1
**ABCB10**	1	1	1	1	1	1	1	1	1	1	1
**ABCB11**	1	1	1	2	1	1	1	1	0	1	1
**ABCC1**	1	1	1	1	1	1	1	1	1	0	1
**ABCC2**	1	1	1	1	1	1	1	1	1	1	1
**ABCC3**	1	1	1	1	1	1	1	1	1	0	0
***ABCC4***	*1*	*1*	*1*	*1*	*2*	*3*	*4*	*4*	*3*	*3*	*1*
**ABCC5**	1	1	(2)	1	1	1	1	1	2	1	0
***ABCC6***	*1*	*1*	*1*	*3*	*1*	*1*	*2*	*2*	*1*	*1*	*1*
**ABCC7**	1	1	1	1	0	0	0	0	0	1	0
**ABCC8**	1	1	1	3	1	1	1	1	1	1	1
**ABCC9**	1	1	1	1	0	0	0	0	0	0	1
**ABCC10**	1	1	1	1	0	1	1	1	1	1	1
**ABCC11**	**1**	**0**	**0**	**0**	**0**	**0**	**0**	**0**	**0**	**0**	**0**
**ABCC12**	1	1	1	1	0	1	1	1	0	1	0
***ABCC13***	***0***	***0***	***0***	***1***	***0***	***0***	***0***	***0***	***0***	***0***	***0***
**ABCD1**	1	1	1	1	1	1	1	1	1	0	1
**ABCD2**	1	1	1	2	1	1	1	0	1	1	1
**ABCD3**	1	1	(2)	2	1	1	1	1	1	1	1
**ABCD4**	1	1	1	1	1	1	1	1	1	1	1
**ABCE1**	1	1	(2)	1	1	1	1	1	1	3	1
**ABCF1**	1	1	1	1	1	1	1	3	1	1	1
**ABCF2**	1	1	(2)	2	1	1	1	1	1	1	1
**ABCF3**	1	1	1	1	1	1	1	1	1	1	1
**ABCG1**	1	1	1	1	1	1	0	0	1	0	1
***ABCG2***	*1*	*1*	*(2)*	*3*	*2*	*2*	*2*	*2*	*2*	*2*	*0*
**ABCG3**	**0**	**1**	**0**	**0**	**0**	**0**	**0**	**0**	**0**	**0**	**0**
***ABCG4***	*1*	*1*	*1*	*2*	*2*	*3*	*2*	*2*	*2*	*1*	*1*
**ABCG5**	1	1	1	1	1	1	1	1	1	1	1
**ABCG8**	1	1	1	1	1	1	1	1	1	1	1
***ABCH1***	***0***	***0***	***0***	***1***	***0***	***0***	***0***	***0***	***0***	***0***	***0***

* Data were based on the Ensembl genome annotation (Release 68), with focus on catfish and other fish species with sequenced genomes. Italicized rows indicate ABC genes that have undergone fish-specific genome duplication; Bolded rows indicate ABC genes that were not found in teleost fish; and the italicized bold rows indicate ABC genes that were not found in any fish except zebrafish. The numbers in parenthesis indicate duplicated ABC genes in catfish.

A previous study reported that ABCB5 genes was absent in non-mammalian genomes [27], but it was identified in both catfish and zebrafish as well as in birds, lizards and *Xenopus* in this study (Figure 5 and Table 2). It’s interesting that ABCC13 was found in the zebrafish genome, which was previously believed to be only in dog and Macaque [8]. Similarly, zebrafish genome harbors ABCH1 that were previously believed to be present only in arthropods [10].

### Orthology and Potential for Functional Inferences of ABC Transporters in Catfish

The ABC transporters play important roles in various physiological processes, with a large portion of which involved in diverse human genetic diseases [8]. Extensive functional studies have been performed in human, but specific functions in fish are largely unknown. The establishment of orthologies should provide potential for functional inferences with the information from model species, with the understanding that the function of lineage-specific genes can be distinct depending on living environments under different selection pressure. Functional inferences for ABC genes that have undergone duplications or losses in teleost fish are of most interest because they are potentially underlying the adaptations to aquatic environments.

In mammals, the ABCA1 gene is required for cholesterol transport from peripheral cells into high-density lipoproteins particles [40]–[42], and the expression of ABCA4 is specific to the photoreceptor cells and is proposed to facilitate the transport of retinoid-lipid complexes out of these cells [43], [44]. The duplication and retention of these two genes in fish species deserve further study.

ABCB1 has broad substrate specificity with one of various roles to remove toxic metabolites and xenobiotics from cells into urine, bile and the intestinal lumen [8]. The broad substrate specificity of ABCB1 may suggest its important roles in wide spectrum of organisms. The ABCB2 (TAP1) and ABCB3 (TAP2) are transporters associated with antigen processing. The TAPs transport peptides derived from proteasomal degradation from the cytosol into the endoplasmic reticulum (ER), which then form complexes with HLA class I molecules for presentation on the cell surface [45]–[47]. ABCB4 is the liver-specific transporter of a mammalian bile component, phosphatidylcholine [27]. The absence of ABCB4-like genes suggests the absence of phospholipids in the bile of teleost fish [8]. The function of the ABCB5 gene is unknown, but it is highly expressed in melanocytes [27]. The human ABCB6 has been described as a mitochondrial porphyrin transporter essential for heme biosynthesis [48], [49], protecting against arsenic cytotoxicity [50] and phenylhydrazine toxicity [51]. The duplication of ABCB6 in catfish and other fish species may confer its detoxification roles in fish.

The high levels of conservation of ABCC subfamily genes indicated that the functions of ABCC genes are critical to a broad spectrum of organisms. Most ABCC genes are involved in the cellular export of toxic compounds. The MRP4/ABCC4 gene has been reported to encode a novel apical organicanion transporter in human kidney proximal tubules [52]. The ABCC7/CFTR gene encodes for a chloride channel that is mutated in patients with cystic fibrosis [53]. The ABCC8 and ABCC9 are receptors for the hypoglycemia drug sulfonylurea and regulation of potassium channels [54]–[56].

ABCE proteins are inhibitors of RNase L [57], and are recently found to play roles in translation initiation in yeast [58] and *C. elegans* [59]. ABCF proteins are involved in ribosome assembly and protein translation [60]. The human ABCG1 and ABCG4 are involved in the export of cholesterol and phospholipids from macrophages to high density lipoproteins [61]. The ABCG5 and ABCG8 are shown to mediate the intestinal and biliary efflux of cholesterol, plant sterols and shellfish sterols [62]. The ABCG2 was first identified in cells from placentas and breast cancers as a multidrug resistance gene. Except for the role in cancer, ABCG2 plays a major role in restricted intestinal absorption of pharmaceuticals, the defense of organs against xenobiotics [63], the protection of haematopoietic stem cells against heme-induced toxicity [64] and the export of urate from kidney [65]. Therefore, the expansion and retention of ABCG2 gene in fish deserves further study.

### Conclusion

A total of 50 ABC transporters were identified from catfish. Phylogenetic analysis, along with syntenic analysis if necessary, allowed annotation of these transporters. While the vast majority of ABC transporters were well conserved through evolution, identification and phylogenetic analysis of ABC transporters in catfish has revealed interesting features of this important group of membrane proteins in fish species: 1) Eleven ABC transporters have not been identified from any fish genomes characterized to date: ABCA6, 8, 9, 10 13, 14, 15, 17, ABCB4, ABCC11, and ABCG3, suggesting their absence from the teleost genomes; 2) In contrast, seven ABC transporters are duplicated in the teleost genomes. They include ABCA1, ABCA4, ABCB6, ABCC4, ABCC6, ABCG2, and ABCG4; and 3) A couple of ABC transporters were found only in zebrafish among teleost genomes characterized to date, and they include ABCC13 and ABCH1 (Table 2).

The high level of conservation of ABC proteins involved in fundamental physiological processes suggests an evolutionary ancestral origin of these proteins. Clear orthologous relationships were established for the majority of ABC genes, enabling the possibility for functional inference of the catfish transporters. However, specific gene duplications were observed in catfish as well as in other fish species. Further research is warranted to unravel the philological significance of gene duplications in adaptation to diverse aquatic environments in fish. The whole set of ABC transporters provide the essential genomic resources for future biochemical, toxicological and physiological studies in catfish.

## Materials and Methods

### Identification of ABC Transporter Genes and Homologs

The ABC transporter genes in catfish were mainly identified through mining a RNA-Seq assembly from our previous study [4] and all catfish genomic resources when necessary [1]–[3], [5], [6]. The RNA-Seq assembly was generated from a transcriptome sequencing of a doubled haploid channel catfish which harbors two identical sets of chromosomes. Therefore, the transcripts were efficiently and accurately assembled because of no allelic variations [4]. All available ABC transporters of human (*Homo sapiens*) and zebrafish (*Danio rerio*) retrieved from GenBank (NCBI) were used as queries to search against the RNA-Seq transcriptome assembly database by standalone TBLASTN (available from NCBI). Same searches were also conducted against the catfish whole genome assembly which was also generated from a doubled haploid channel catfish (unpublished data) to confirm sequence accuracy. The coding sequences from retrieved RNA-Seq transcripts were predicted using getorf from the EMBOSS tools, and validated by BLASTP against NCBI non-redundant protein sequence database (nr). The retrieved genome sequences were subjected to *ab initio* gene prediction by GENSCAN [66]. The complete coding sequences were confirmed by comparing with homologous proteins of zebrafish and/or human from NCBI RefSeq protein database. The simple modular architecture research tool (SMART) was used to predict the conserved domains based on sequence homology and further confirmed by conserved domain prediction from BLAST. The full-length amino acid sequences as well as the partial sequences coding for the conserved domains were used in the phylogenetic analysis. The ABC proteins from other organisms were retrieved from the Ensembl genome database (Release 68) for phylogenetic analysis with exclusion of partial sequences.

### Phylogenetic and Orthology Analysis

In order to annotate the ABC transporters, phylogenetic analysis was conducted with reference ABC proteins from zebrafish and human, and other representative vertebrate species. For nomenclatures of catfish ABCs, whenever possible we followed those of zebrafish because zebrafish is the most closely related model species to catfish, and its genome went through a third round of whole genome duplication. Multiple protein sequences were aligned by ClustalW [67], Muscle v3.8 [68] and the L-INS-i, E-INS-i and G-INS-i methods from MAFFT v7.01 [69], with default parameters. We employed the program MUMSA [70] to select the best-scoring multiple alignment. The alignment with best quality was then curated using Gblocks to eliminate poorly aligned positions and divergent regions [71]. The best model of protein evolution was determined by ProtTest according to the Bayesian information criterion [72]. The best-fit model was the JTT+I+G model which uses a Jones-Taylor-Thornton (JTT) matrix and incorporates a proportion of invariant sites (+I) and the gamma distribution for modeling rate heterogeneity (+G). The phylogenetic trees were reconstructed with maximum likelihood, minimum evolution and maximum parsimony. We performed maximum likelihood analysis in RAxML 7.3.6 [73] and MEGA5 [74] with bootstrap test of 1000 replicates. The minimum evolution and maximum parsimony trees were constructed using MEGA5 with the Close-Neighbor-Interchange (CNI) heuristic and Subtree-Pruning-Regrafting (SPR) methods, and bootstrap test of 1000 replicates, respectively. We manually checked and combined the consensus trees from different methods using TreeGraph2 [75]. The subfamily assignment of catfish ABC proteins was determined by phylogenetic analysis with ABC transporter proteins from zebrafish and human. Separate phylogenetic analyses were constructed per subfamily using the same methodology with other representative vertebrate species including zebrafish, medaka, fugu, *Tetraodon*, stickleback, tilapia, cod, coelacanth, lamprey, chicken/turkey, *Xenopus*, lizard, mouse and human (Table S1). The ortholgoy analysis was conducted by analyzing synteny regions harboring ABCB from several vertebrates based on genome information from Ensembl (Release 68). Molecular clock hypothesis was tested using LINTREE [76].

## Supporting Information

Figure S1 **Functional domain organizations of catfish ABC transporters.**
(PDF)

Figure S2 **Phylogenetic trees of catfish ABC transporters with reference ABC transporters from all other species.**
(PDF)

Table S1 **Gene names and accessions of reference ABC transporters used in this study.**
(XLSX)
