# Supplementary figures and images for: Genome-Wide Identification, Characterization and Phylogenetic Analysis of 50 Catfish ATP-Binding Cassette (ABC) Transporter Genes
Source: PLoS One. 2013 May 16;8(5):e63895. doi: 10.1371/journal.pone.0063895 (PMC3655950; doi:10.1371/journal.pone.0063895)

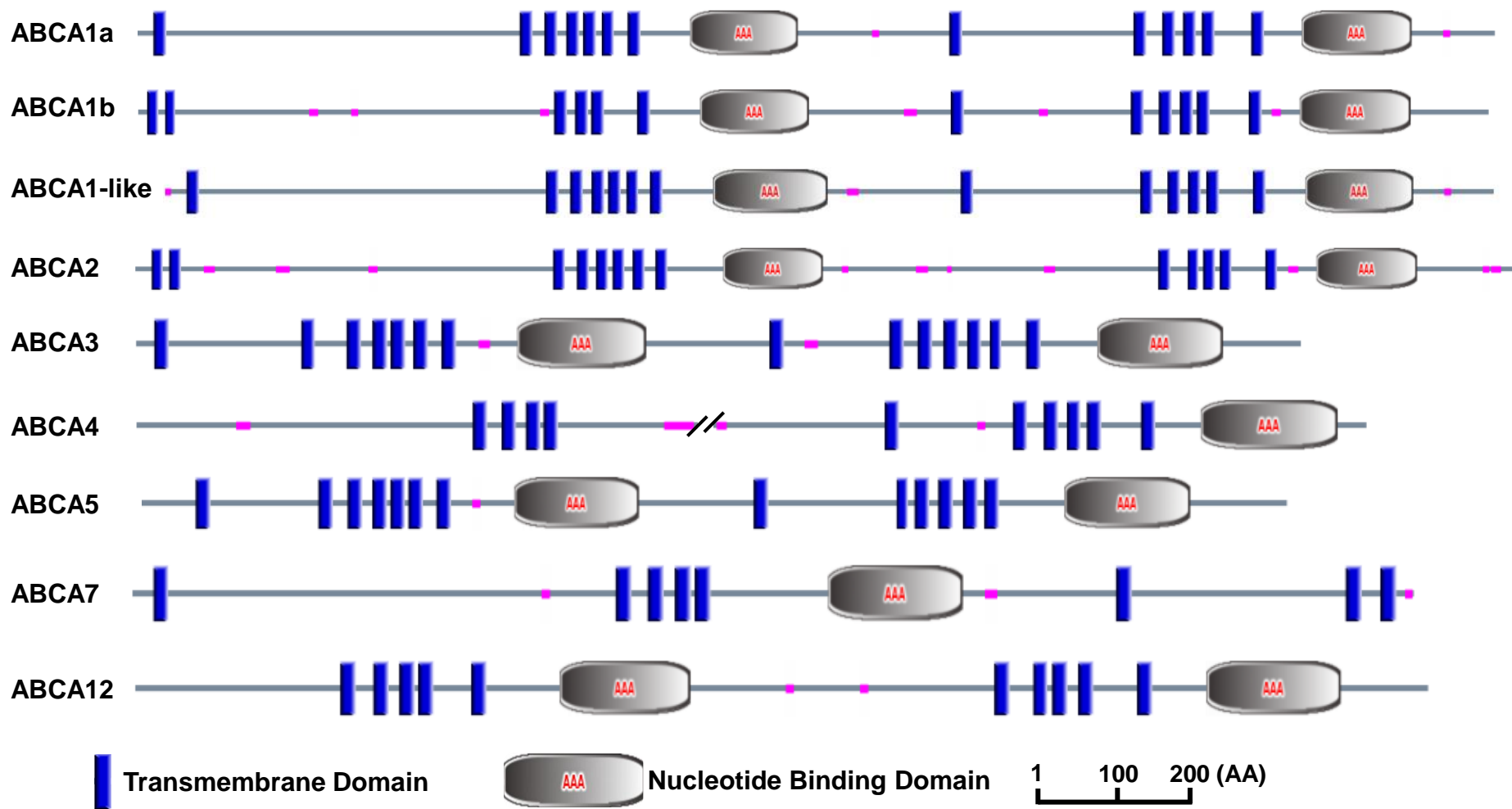

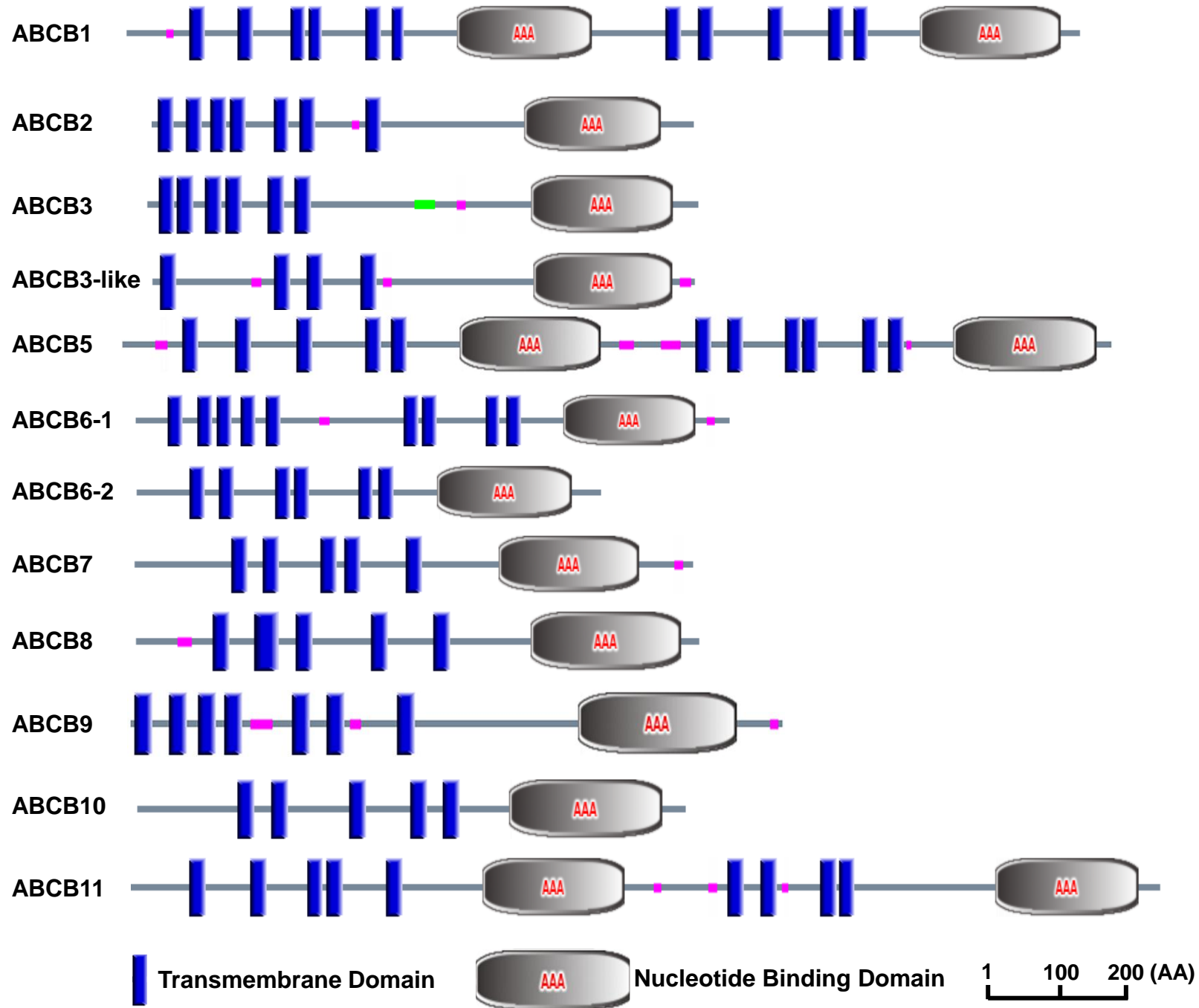

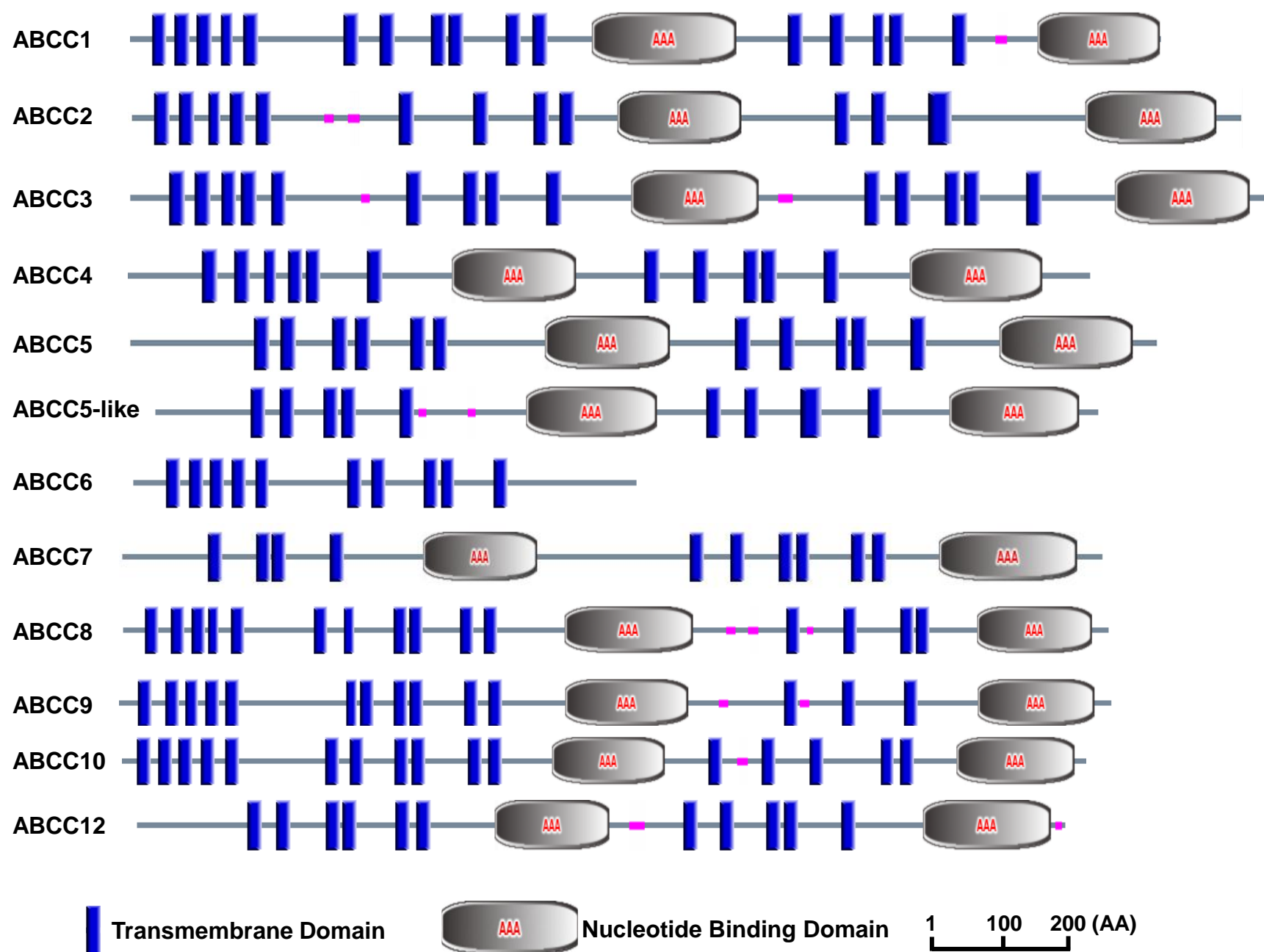

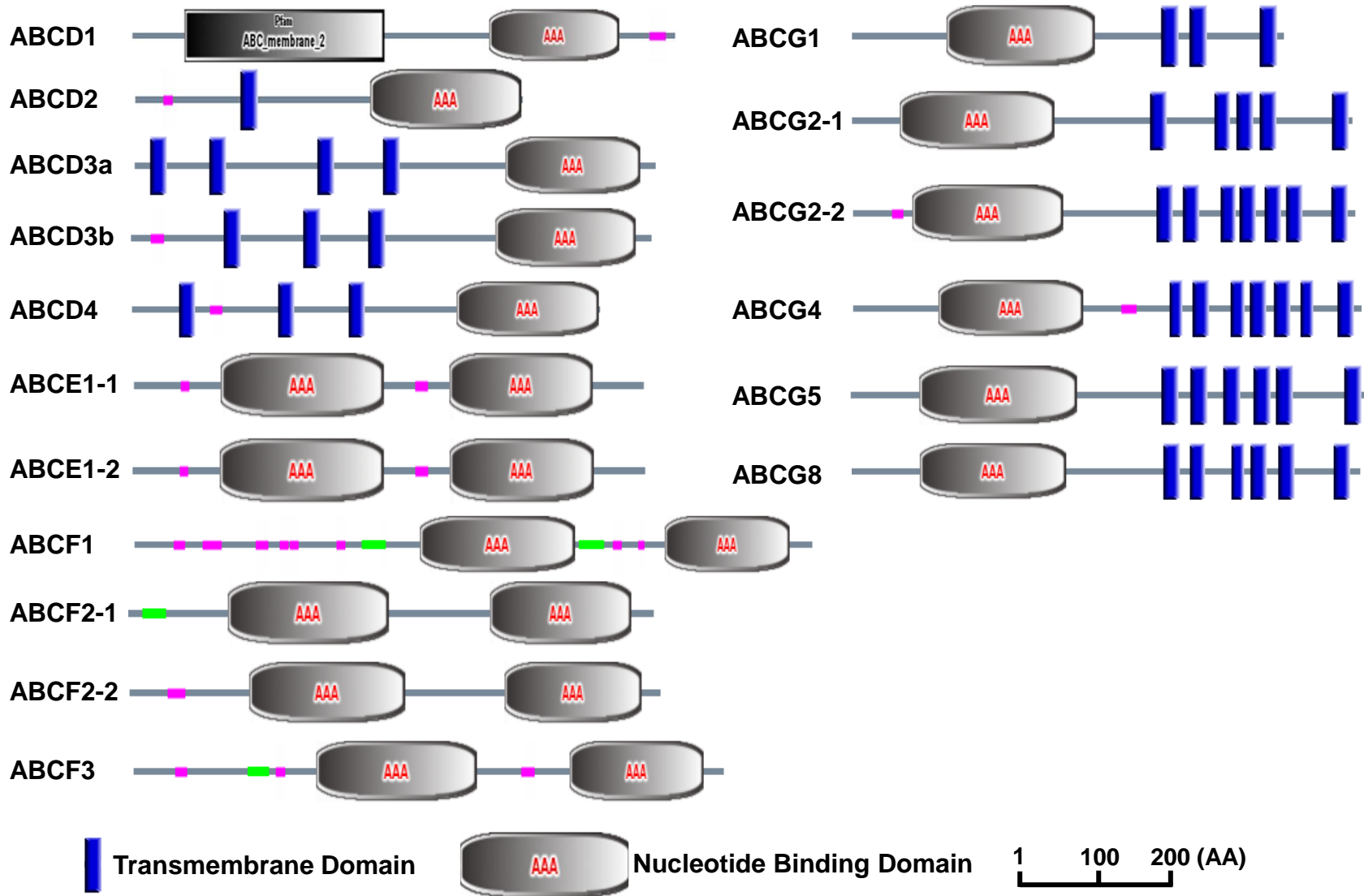

Supplement: Figure S1 — Functional domain organizations of catfish ABC transporters. (PDF) [file pone.0063895.s001.pdf]
