# Supplementary material for: Genome-Wide Identification, Characterization and Phylogenetic Analysis of 50 Catfish ATP-Binding Cassette (ABC) Transporter Genes
Source: PLoS One. 2013 May 16;8(5):e63895. doi: 10.1371/journal.pone.0063895 (PMC3655950; doi:10.1371/journal.pone.0063895)
Supplement: Figure S2 — Phylogenetic trees of catfish ABC transporters with reference ABC transporters from all other species. (PDF) [file pone.0063895.s002.pdf]

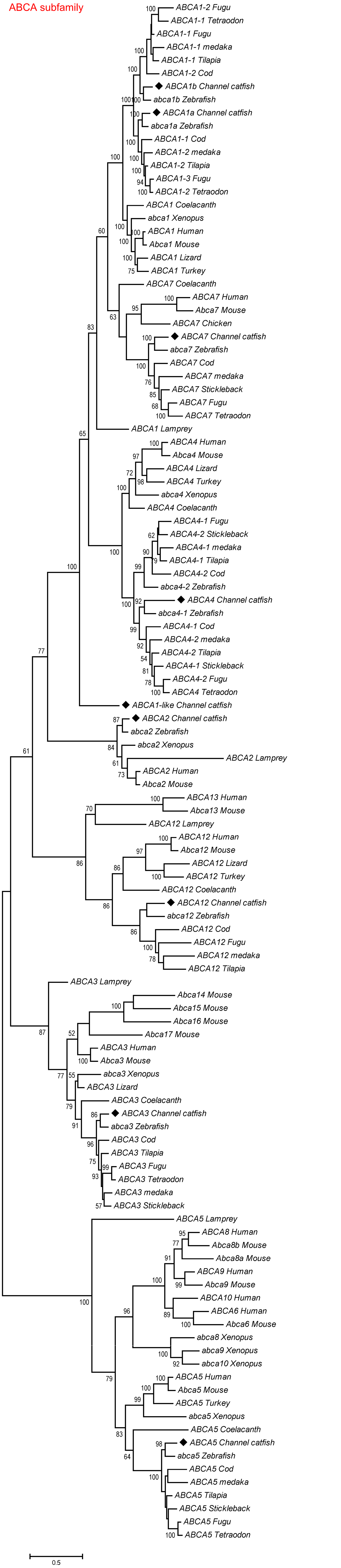
ABCA subfamily
ABCA1-2 Fugu
ABCA1-1 Tetraodon
ABCA1-1 Fugu
ABCA1-1 medaka
ABCA1-1 Tilapia
ABCA1-2 Cod
ABCA1b Channel catfish
abca1b Zebrafish
ABCA1a Channel catfish
abca1a Zebrafish
ABCA1-1 Cod
ABCA1-2 medaka
ABCA1-2 Tilapia
ABCA1-3 Fugu
ABCA1-2 Tetraodon
ABCA1 Coelacanth
abca1 Xenopus
ABCA1 Human
Abca1 Mouse
ABCA1 Lizard
ABCA1 Turkey
ABCA7 Coelacanth
ABCA7 Human
Abca7 Mouse
ABCA7 Chicken
ABCA7 Channel catfish
abca7 Zebrafish
ABCA7 Cod
ABCA7 medaka
ABCA7 Stickleback
ABCA7 Fugu
ABCA7 Tetraodon
ABCA1 Lamprey
ABCA4 Human
Abca4 Mouse
ABCA4 Lizard
ABCA4 Turkey
abca4 Xenopus
ABCA4 Coelacanth
ABCA4-1 Fugu
ABCA4-2 Stickleback
ABCA4-1 medaka
ABCA4-1 Tilapia
ABCA4-2 Cod
abca4-2 Zebrafish
ABCA4 Channel catfish
abca4-1 Zebrafish
ABCA4-1 Cod
ABCA4-2 medaka
ABCA4-2 Tilapia
ABCA4-1 Stickleback
ABCA4-2 Fugu
ABCA4 Tetraodon
ABCA1-like Channel catfish
ABCA2 Channel catfish
abca2 Zebrafish
abca2 Xenopus
ABCA2 Lamprey
ABCA2 Human
Abca2 Mouse
ABCA13 Human
Abca13 Mouse
ABCA12 Lamprey
ABCA12 Human
Abca12 Mouse
ABCA12 Lizard
ABCA12 Turkey
ABCA12 Coelacanth
ABCA12 Channel catfish
abca12 Zebrafish
ABCA12 Cod
ABCA12 Fugu
ABCA12 medaka
ABCA12 Tilapia
ABCA3 Lamprey
Abca14 Mouse
Abca15 Mouse
Abca16 Mouse
Abca17 Mouse
ABCA3 Human
Abca3 Mouse
abca3 Xenopus
ABCA3 Lizard
ABCA3 Coelacanth
ABCA3 Channel catfish
abca3 Zebrafish
ABCA3 Cod
ABCA3 Tilapia
ABCA3 Fugu
ABCA3 Tetraodon
ABCA3 medaka
ABCA3 Stickleback
ABCA5 Lamprey
ABCA8 Human
Abca8b Mouse
Abca8a Mouse
ABCA9 Human
Abca9 Mouse
ABCA10 Human
ABCA6 Human
Abca6 Mouse
abca8 Xenopus
abca9 Xenopus
abca10 Xenopus
ABCA5 Human
Abca5 Mouse
ABCA5 Turkey
abca5 Xenopus
ABCA5 Coelacanth
ABCA5 Channel catfish
abca5 Zebrafish
ABCA5 Cod
ABCA5 medaka
ABCA5 Tilapia
ABCA5 Stickleback
ABCA5 Fugu
ABCA5 Tetraodon
0.5

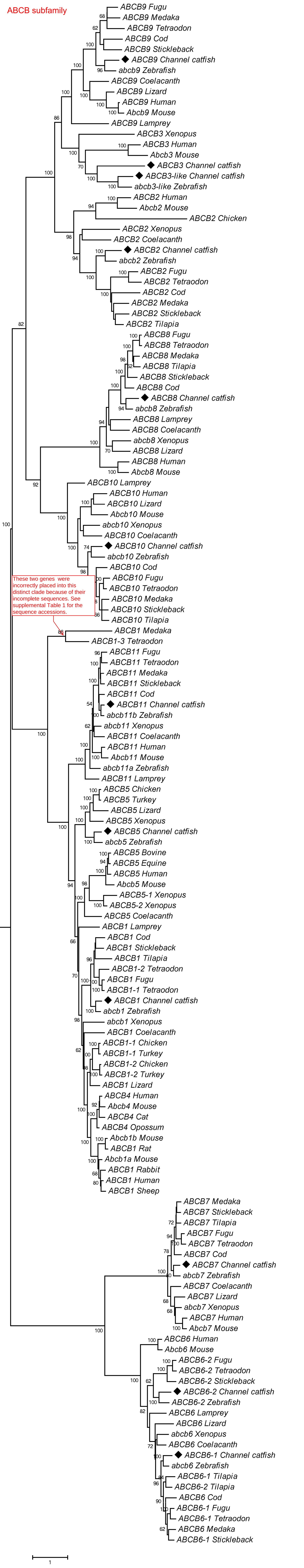

ABCB subfamily
ABCB9 Fugu
ABCB9 Medaka
ABCB9 Tetraodon
ABCB9 Cod
ABCB9 Stickleback
ABCB9 Channel catfish
abcb9 Zebrafish
ABCB9 Coelacanth
ABCB9 Lizard
ABCB9 Human
Abcb9 Mouse
ABCB9 Lamprey
ABCB3 Xenopus
ABCB3 Human
Abcb3 Mouse
ABCB3 Channel catfish
ABCB3-like Channel catfish
abcb3-like Zebrafish
ABCB2 Human
Abcb2 Mouse
ABCB2 Chicken
ABCB2 Xenopus
ABCB2 Coelacanth
ABCB2 Channel catfish
abcb2 Zebrafish
ABCB2 Fugu
ABCB2 Tetraodon
ABCB2 Cod
ABCB2 Medaka
ABCB2 Stickleback
ABCB2 Tilapia
ABCB8 Fugu
ABCB8 Tetraodon
ABCB8 Medaka
ABCB8 Tilapia
ABCB8 Stickleback
ABCB8 Cod
ABCB8 Channel catfish
abcb8 Zebrafish
ABCB8 Lamprey
ABCB8 Coelacanth
abcb8 Xenopus
ABCB8 Lizard
ABCB8 Human
Abcb8 Mouse
ABCB10 Lamprey
ABCB10 Human
ABCB10 Lizard
Abcb10 Mouse
abcb10 Xenopus
ABCB10 Coelacanth
ABCB10 Channel catfish
abcb10 Zebrafish
ABCB10 Cod
ABCB10 Fugu
ABCB10 Tetraodon
ABCB10 Medaka
ABCB10 Stickleback
ABCB10 Tilapia
These two genes were incorrectly placed into this distinct clade because of their incomplete sequences. See supplemental Table 1 for the sequence accessions.
ABCB1 Medaka
ABCB1-3 Tetraodon
ABCB11 Fugu
ABCB11 Tetraodon
ABCB11 Medaka
ABCB11 Stickleback
ABCB11 Cod
ABCB11 Channel catfish
abcb11b Zebrafish
abcb11 Xenopus
ABCB11 Coelacanth
ABCB11 Human
Abcb11 Mouse
abcb11a Zebrafish
ABCB11 Lamprey
ABCB5 Chicken
ABCB5 Turkey
ABCB5 Lizard
ABCB5 Xenopus
ABCB5 Channel catfish
abcb5 Zebrafish
ABCB5 Bovine
ABCB5 Equine
ABCB5 Human
Abcb5 Mouse
ABCB5-1 Xenopus
ABCB5-2 Xenopus
ABCB5 Coelacanth
ABCB1 Lamprey
ABCB1 Cod
ABCB1 Stickleback
ABCB1 Tilapia
ABCB1-2 Tetraodon
ABCB1 Fugu
ABCB1-1 Tetraodon
ABCB1 Channel catfish
abcb1 Zebrafish
abcb1 Xenopus
ABCB1 Coelacanth
ABCB1-1 Chicken
ABCB1-1 Turkey
ABCB1-2 Chicken
ABCB1-2 Turkey
ABCB1 Lizard
ABCB4 Human
Abcb4 Mouse
ABCB4 Cat
ABCB4 Opossum
Abcb1b Mouse
ABCB1 Rat
Abcb1a Mouse
ABCB1 Rabbit
ABCB1 Human
ABCB1 Sheep
ABCB7 Medaka
ABCB7 Stickleback
ABCB7 Tilapia
ABCB7 Fugu
ABCB7 Tetraodon
ABCB7 Cod
ABCB7 Channel catfish
abcb7 Zebrafish
ABCB7 Coelacanth
ABCB7 Lizard
abcb7 Xenopus
ABCB7 Human
Abcb7 Mouse
ABCB6 Human
Abcb6 Mouse
ABCB6-2 Fugu
ABCB6-2 Tetraodon
ABCB6-2 Stickleback
ABCB6-2 Channel catfish
ABCB6-2 Zebrafish
ABCB6 Lamprey
ABCB6 Lizard
abcb6 Xenopus
ABCB6 Coelacanth
ABCB6-1 Channel catfish
abcb6 Zebrafish
ABCB6-1 Tilapia
ABCB6-2 Tilapia
ABCB6 Cod
ABCB6-1 Fugu
ABCB6-1 Tetraodon
ABCB6 Medaka
ABCB6-1 Stickleback
1

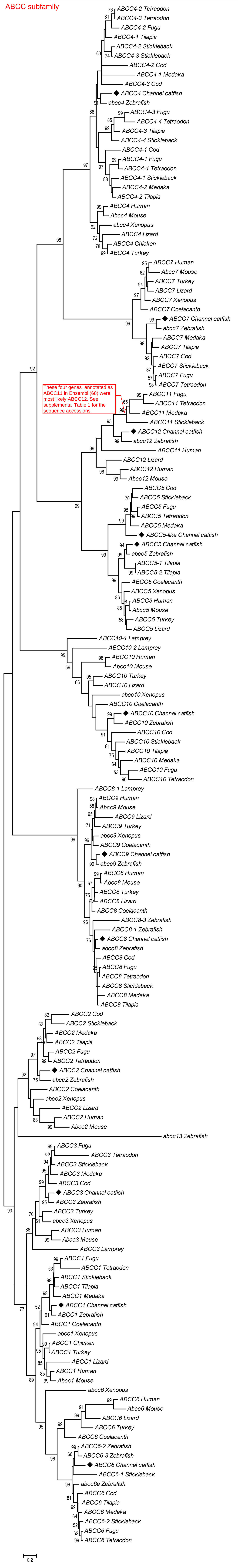
ABCC subfamily
ABCC4-2 Tetraodon
ABCC4-3 Tetraodon
ABCC4-2 Fugu
ABCC4-1 Tilapia
ABCC4-2 Stickleback
ABCC4-3 Stickleback
ABCC4-2 Cod
ABCC4-1 Medaka
ABCC4-3 Cod
ABCC4 Channel catfish
abcc4 Zebrafish
ABCC4-3 Fugu
ABCC4-4 Tetraodon
ABCC4-3 Tilapia
ABCC4-4 Stickleback
ABCC4-1 Cod
ABCC4-1 Fugu
ABCC4-1 Tetraodon
ABCC4-1 Stickleback
ABCC4-2 Medaka
ABCC4-2 Tilapia
ABCC4 Human
Abcc4 Mouse
abcc4 Xenopus
ABCC4 Lizard
ABCC4 Chicken
ABCC4 Turkey
ABCC7 Human
Abcc7 Mouse
ABCC7 Turkey
ABCC7 Lizard
ABCC7 Xenopus
ABCC7 Coelacanth
ABCC7 Channel catfish
abcc7 Zebrafish
ABCC7 Medaka
ABCC7 Tilapia
ABCC7 Cod
ABCC7 Stickleback
ABCC7 Fugu
ABCC7 Tetraodon
These four genes annotated as ABCC11 in Ensembl (68) were most likely ABCC12. See supplemental Table 1 for the sequence accessions.
ABCC11 Fugu
ABCC11 Tetraodon
ABCC11 Medaka
ABCC11 Stickleback
ABCC12 Channel catfish
abcc12 Zebrafish
ABCC11 Human
ABCC12 Lizard
ABCC12 Human
Abcc12 Mouse
ABCC5 Cod
ABCC5 Stickleback
ABCC5 Fugu
ABCC5 Tetraodon
ABCC5 Medaka
ABCC5-like Channel catfish
ABCC5 Channel catfish
abcc5 Zebrafish
ABCC5-1 Tilapia
ABCC5-2 Tilapia
ABCC5 Coelacanth
ABCC5 Xenopus
ABCC5 Human
Abcc5 Mouse
ABCC5 Turkey
ABCC5 Lizard
ABCC10-1 Lamprey
ABCC10-2 Lamprey
ABCC10 Human
Abcc10 Mouse
ABCC10 Turkey
ABCC10 Lizard
abcc10 Xenopus
ABCC10 Coelacanth
ABCC10 Channel catfish
ABCC10 Zebrafish
ABCC10 Cod
ABCC10 Stickleback
ABCC10 Tilapia
ABCC10 Medaka
ABCC10 Fugu
ABCC10 Tetraodon
ABCC8-1 Lamprey
ABCC9 Human
Abcc9 Mouse
ABCC9 Lizard
ABCC9 Turkey
abcc9 Xenopus
ABCC9 Coelacanth
ABCC9 Channel catfish
abcc9 Zebrafish
ABCC8 Human
Abcc8 Mouse
ABCC8 Turkey
ABCC8 Lizard
ABCC8 Coelacanth
ABCC8-3 Zebrafish
ABCC8-1 Zebrafish
ABCC8 Channel catfish
abcc8 Zebrafish
ABCC8 Cod
ABCC8 Fugu
ABCC8 Tetraodon
ABCC8 Stickleback
ABCC8 Medaka
ABCC8 Tilapia
ABCC2 Cod
ABCC2 Stickleback
ABCC2 Medaka
ABCC2 Tilapia
ABCC2 Fugu
ABCC2 Tetraodon
ABCC2 Channel catfish
abcc2 Zebrafish
ABCC2 Coelacanth
abcc2 Xenopus
ABCC2 Lizard
ABCC2 Human
Abcc2 Mouse
abcc13 Zebrafish
ABCC3 Fugu
ABCC3 Tetraodon
ABCC3 Stickleback
ABCC3 Medaka
ABCC3 Cod
ABCC3 Channel catfish
ABCC3 Zebrafish
ABCC3 Turkey
abcc3 Xenopus
ABCC3 Human
Abcc3 Mouse
ABCC3 Lamprey
ABCC1 Fugu
ABCC1 Tetraodon
ABCC1 Stickleback
ABCC1 Tilapia
ABCC1 Medaka
ABCC1 Channel catfish
ABCC1 Zebrafish
ABCC1 Coelacanth
abcc1 Xenopus
ABCC1 Chicken
ABCC1 Turkey
ABCC1 Lizard
ABCC1 Human
Abcc1 Mouse
abcc6 Xenopus
ABCC6 Human
Abcc6 Mouse
ABCC6 Lizard
ABCC6 Turkey
ABCC6 Coelacanth
ABCC6-2 Zebrafish
ABCC6-3 Zebrafish
ABCC6 Channel catfish
ABCC6-1 Stickleback
abcc6a Zebrafish
ABCC6 Cod
ABCC6 Tilapia
ABCC6 Medaka
ABCC6-2 Stickleback
ABCC6 Fugu
ABCC6 Tetraodon
0.2

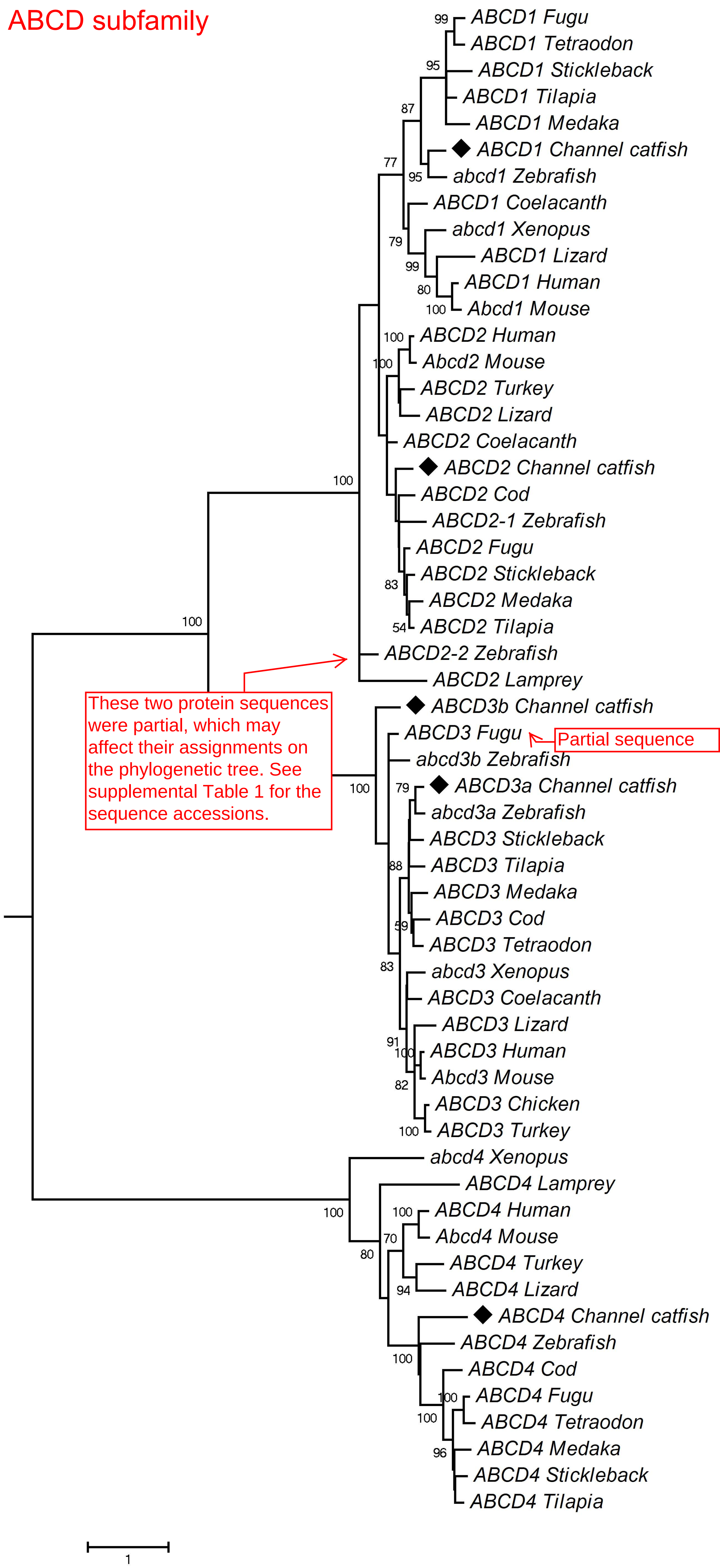
ABCD subfamily
ABCD1 Fugu
ABCD1 Tetraodon
ABCD1 Stickleback
ABCD1 Tilapia
ABCD1 Medaka
ABCD1 Channel catfish
abcd1 Zebrafish
ABCD1 Coelacanth
abcd1 Xenopus
ABCD1 Lizard
ABCD1 Human
Abcd1 Mouse
ABCD2 Human
Abcd2 Mouse
ABCD2 Turkey
ABCD2 Lizard
ABCD2 Coelacanth
ABCD2 Channel catfish
ABCD2 Cod
ABCD2-1 Zebrafish
ABCD2 Fugu
ABCD2 Stickleback
ABCD2 Medaka
ABCD2 Tilapia
ABCD2-2 Zebrafish
ABCD2 Lamprey
These two protein sequences were partial, which may affect their assignments on the phylogenetic tree. See supplemental Table 1 for the sequence accessions.
ABCD3b Channel catfish
ABCD3 Fugu
Partial sequence
abcd3b Zebrafish
ABCD3a Channel catfish
abcd3a Zebrafish
ABCD3 Stickleback
ABCD3 Tilapia
ABCD3 Medaka
ABCD3 Cod
ABCD3 Tetraodon
abcd3 Xenopus
ABCD3 Coelacanth
ABCD3 Lizard
ABCD3 Human
Abcd3 Mouse
ABCD3 Chicken
ABCD3 Turkey
abcd4 Xenopus
ABCD4 Lamprey
ABCD4 Human
Abcd4 Mouse
ABCD4 Turkey
ABCD4 Lizard
ABCD4 Channel catfish
ABCD4 Zebrafish
ABCD4 Cod
ABCD4 Fugu
ABCD4 Tetraodon
ABCD4 Medaka
ABCD4 Stickleback
ABCD4 Tilapia
1

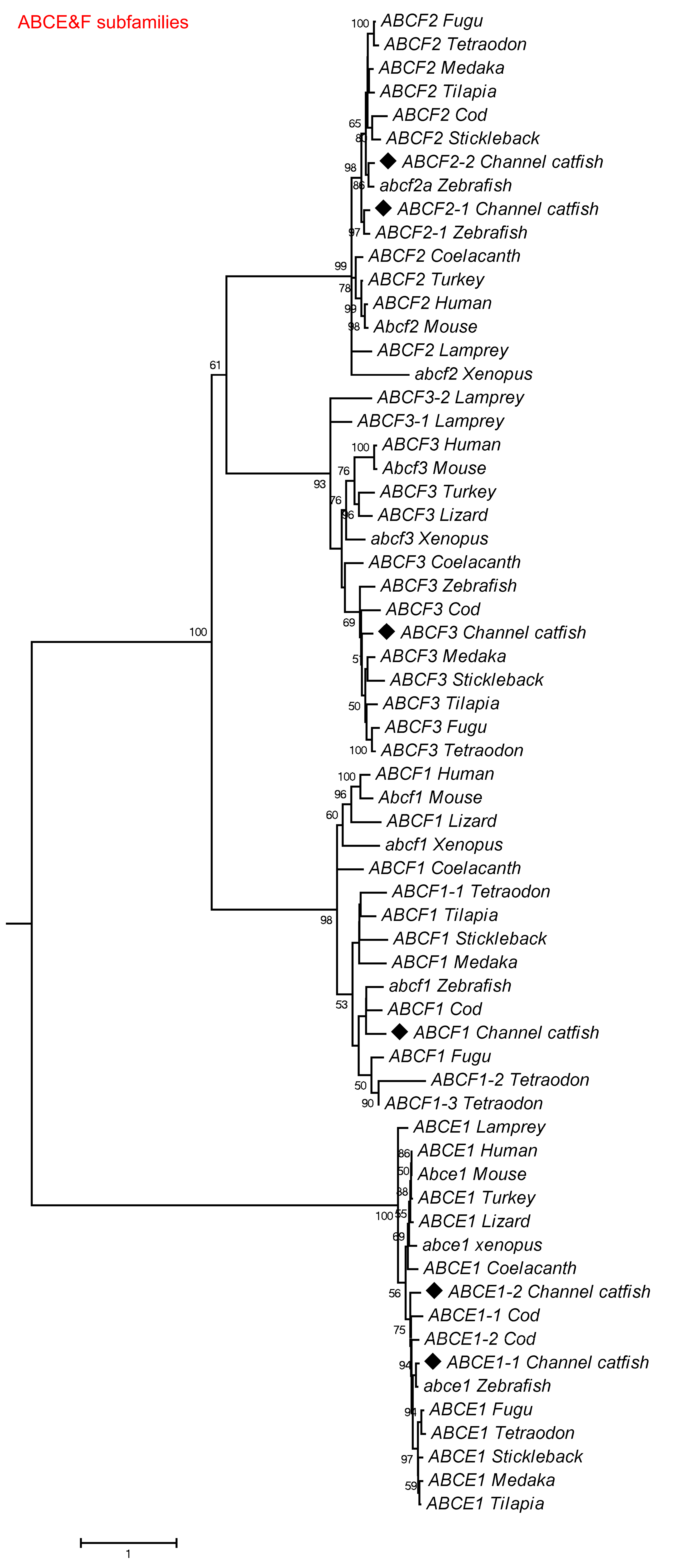
ABCE&F subfamilies
ABCF2 Fugu
ABCF2 Tetraodon
ABCF2 Medaka
ABCF2 Tilapia
ABCF2 Cod
ABCF2 Stickleback
ABCF2-2 Channel catfish
abcf2a Zebrafish
ABCF2-1 Channel catfish
ABCF2-1 Zebrafish
ABCF2 Coelacanth
ABCF2 Turkey
ABCF2 Human
Abcf2 Mouse
ABCF2 Lamprey
abcf2 Xenopus
ABCF3-2 Lamprey
ABCF3-1 Lamprey
ABCF3 Human
Abcf3 Mouse
ABCF3 Turkey
ABCF3 Lizard
abcf3 Xenopus
ABCF3 Coelacanth
ABCF3 Zebrafish
ABCF3 Cod
ABCF3 Channel catfish
ABCF3 Medaka
ABCF3 Stickleback
ABCF3 Tilapia
ABCF3 Fugu
ABCF3 Tetraodon
ABCF1 Human
Abcf1 Mouse
ABCF1 Lizard
abcf1 Xenopus
ABCF1 Coelacanth
ABCF1-1 Tetraodon
ABCF1 Tilapia
ABCF1 Stickleback
ABCF1 Medaka
abcf1 Zebrafish
ABCF1 Cod
ABCF1 Channel catfish
ABCF1 Fugu
ABCF1-2 Tetraodon
ABCF1-3 Tetraodon
ABCE1 Lamprey
ABCE1 Human
Abce1 Mouse
ABCE1 Turkey
ABCE1 Lizard
abce1 xenopus
ABCE1 Coelacanth
ABCE1-2 Channel catfish
ABCE1-1 Cod
ABCE1-2 Cod
ABCE1-1 Channel catfish
abce1 Zebrafish
ABCE1 Fugu
ABCE1 Tetraodon
ABCE1 Stickleback
ABCE1 Medaka
ABCE1 Tilapia
1

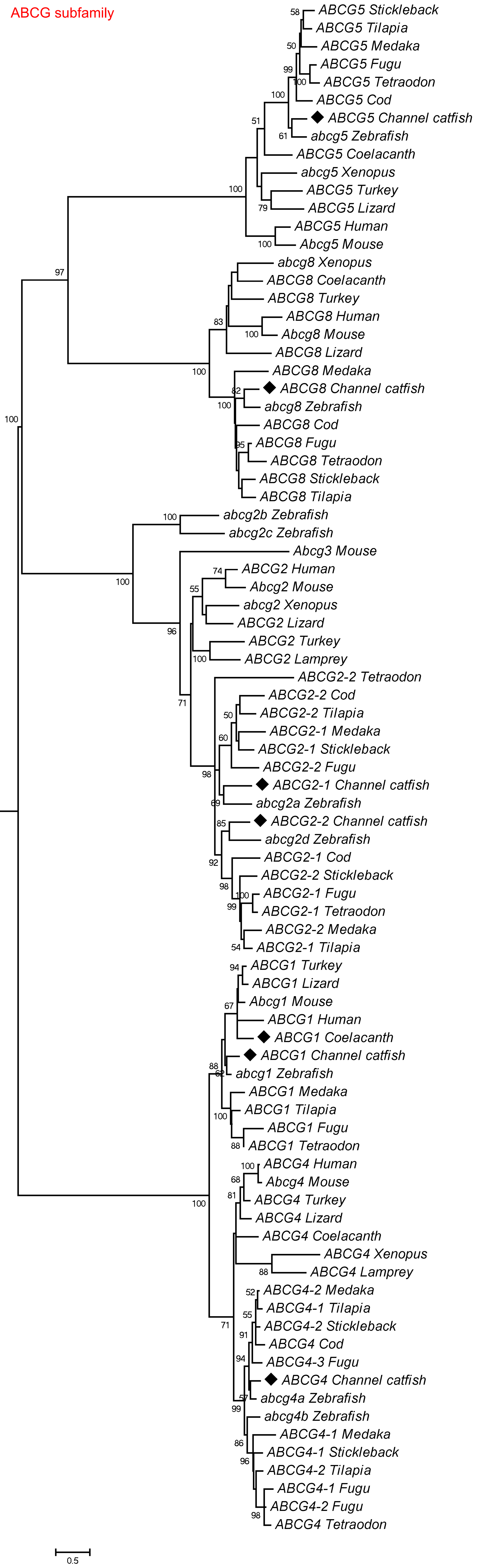
ABCG subfamily
ABCG5 Stickleback
ABCG5 Tilapia
ABCG5 Medaka
ABCG5 Fugu
ABCG5 Tetraodon
ABCG5 Cod
ABCG5 Channel catfish
abcg5 Zebrafish
ABCG5 Coelacanth
abcg5 Xenopus
ABCG5 Turkey
ABCG5 Lizard
ABCG5 Human
Abcg5 Mouse
abcg8 Xenopus
ABCG8 Coelacanth
ABCG8 Turkey
ABCG8 Human
Abcg8 Mouse
ABCG8 Lizard
ABCG8 Medaka
ABCG8 Channel catfish
abcg8 Zebrafish
ABCG8 Cod
ABCG8 Fugu
ABCG8 Tetraodon
ABCG8 Stickleback
ABCG8 Tilapia
abcg2b Zebrafish
abcg2c Zebrafish
Abcg3 Mouse
ABCG2 Human
Abcg2 Mouse
abcg2 Xenopus
ABCG2 Lizard
ABCG2 Turkey
ABCG2 Lamprey
ABCG2-2 Tetraodon
ABCG2-2 Cod
ABCG2-2 Tilapia
ABCG2-1 Medaka
ABCG2-1 Stickleback
ABCG2-2 Fugu
ABCG2-1 Channel catfish
abcg2a Zebrafish
ABCG2-2 Channel catfish
abcg2d Zebrafish
ABCG2-1 Cod
ABCG2-2 Stickleback
ABCG2-1 Fugu
ABCG2-1 Tetraodon
ABCG2-2 Medaka
ABCG2-1 Tilapia
ABCG1 Turkey
ABCG1 Lizard
Abcg1 Mouse
ABCG1 Human
ABCG1 Coelacanth
ABCG1 Channel catfish
abcg1 Zebrafish
ABCG1 Medaka
ABCG1 Tilapia
ABCG1 Fugu
ABCG1 Tetraodon
ABCG4 Human
Abcg4 Mouse
ABCG4 Turkey
ABCG4 Lizard
ABCG4 Coelacanth
ABCG4 Xenopus
ABCG4 Lamprey
ABCG4-2 Medaka
ABCG4-1 Tilapia
ABCG4-2 Stickleback
ABCG4 Cod
ABCG4-3 Fugu
ABCG4 Channel catfish
abcg4a Zebrafish
abcg4b Zebrafish
ABCG4-1 Medaka
ABCG4-1 Stickleback
ABCG4-2 Tilapia
ABCG4-1 Fugu
ABCG4-2 Fugu
ABCG4 Tetraodon
0.5
